# Revision and Microtomography of the *Pheidole knowlesi* Group, an Endemic Ant Radiation in Fiji (Hymenoptera, Formicidae, Myrmicinae)

**DOI:** 10.1371/journal.pone.0158544

**Published:** 2016-07-27

**Authors:** Georg Fischer, Eli M. Sarnat, Evan P. Economo

**Affiliations:** 1 Okinawa Institute of Science & Technology Graduate University, 1919-1 Tancha, Onna-son, Okinawa, Japan, 904-0495; 2 Department of Ecology and Evolutionary Biology, University of Michigan, Ann Arbor, MI, United States of America; CNRS, FRANCE

## Abstract

The Fijian islands, a remote archipelago in the southwestern Pacific, are home to a number of spectacular endemic radiations of plants and animals. Unlike most Pacific archipelagos, these evolutionary radiations extend to social insects, including ants. One of the most dramatic examples of ant radiation in Fiji has occurred in the hyperdiverse genus *Pheidole*. Most of the 17 native Fijian *Pheidole* belong to one of two species groups that descended from a single colonization, yet have evolved dramatically contrasting morphologies: the spinescent *P*. *roosevelti* species group, and the more morphologically conservative *P*. *knowlesi* species group. Here we revise the *knowlesi* group, in light of recent phylogenetic results, and enhanced with modern methods of X-ray microtomography. We recognize six species belonging to this group, including two of which we describe as new: *Pheidole caldwelli* Mann, *Pheidole kava* sp. n., *Pheidole knowlesi* Mann, *P*. *ululevu* sp. n., *P*. *vatu* Mann, and *P*. *wilsoni* Mann. Detailed measurements and descriptions, identification keys, and high-resolution images for queens, major and minor workers are provided. In addition, we include highly detailed 3D surface reconstructions for all available castes.

## Introduction

The terrestrial ecosystems of the Fijian archipelago have evolved in relative isolation for millions of years, leading to the evolution of a highly endemic and interesting biota [1,2]. Such island systems have long served as natural laboratories for developing and testing ecological and evolutionary theory [3,4]. They are also a high priority for conservation attention as they harbor some of the most geographically restricted and ecologically threatened lineages on the planet [5,6]. Indeed, Fiji’s terrestrial biota is increasingly threatened by the adverse effects of land-use change, climate change, and the impacts of invasive species [1]. However, much work remains to fully document these island faunas–particularly invertebrate taxa–and reconstruct their evolutionary histories. Toward that end, major efforts have been recently mounted to inventory Fiji’s arthropods [7], and the phylogenetic histories of several groups have been reconstructed [8–12].

Fiji’s ant fauna has a long history of study, and has inspired fundamental contributions to evolutionary biogeography [3,13–16]. It was first treated by Mann in his 1921 monograph [17]. Almost a century later, Sarnat and Economo [2] completed a comprehensive update of the fauna, recognizing 187 species currently classified into 46 genera and 8 subfamilies. Of these, approximately 40 species remain undescribed [2]. The current contribution is the latest in a series of taxonomically-oriented studies intended to fully document this highly endemic and geographically localized fauna. Recently revised genera and species groups include *Lordomyrma* [18,19], *Pristomyrmex* [20], *Proceratium* [21], and the *Pheidole roosevelti* group [22].

The ants of Fiji are of special interest due to their relevance to the taxon cycle hypothesis, which was developed in the same Melanesian ant system [14]. In particular, the evolutionary and ecological relationships between the 19 *Pheidole* species in Fiji have been studied in the context of the taxon cycle [23–25], and generally interpreted to be consistent with the hypothesis. The Fijian *Pheidole* are derived from seven colonizations of the archipelago [23,26]. Two of those are recent human-mediated introductions: the notoriously invasive *Pheidole megacephala* (Fabricius) and *P*. *fervens* Smith [27]. Three represent distantly related taxa that are presumed native to Oceania, but have not diversified in Fiji (*P*. *oceanica* Mayr, *P*. *sexspinosa* Mayr, *P*. *umbonata* Mayr) [25]. One endemic species, *P*. *onifera*, is the sole known descendent of a lineage colonizing from New Guinea or Australia.

**Fig 1:**
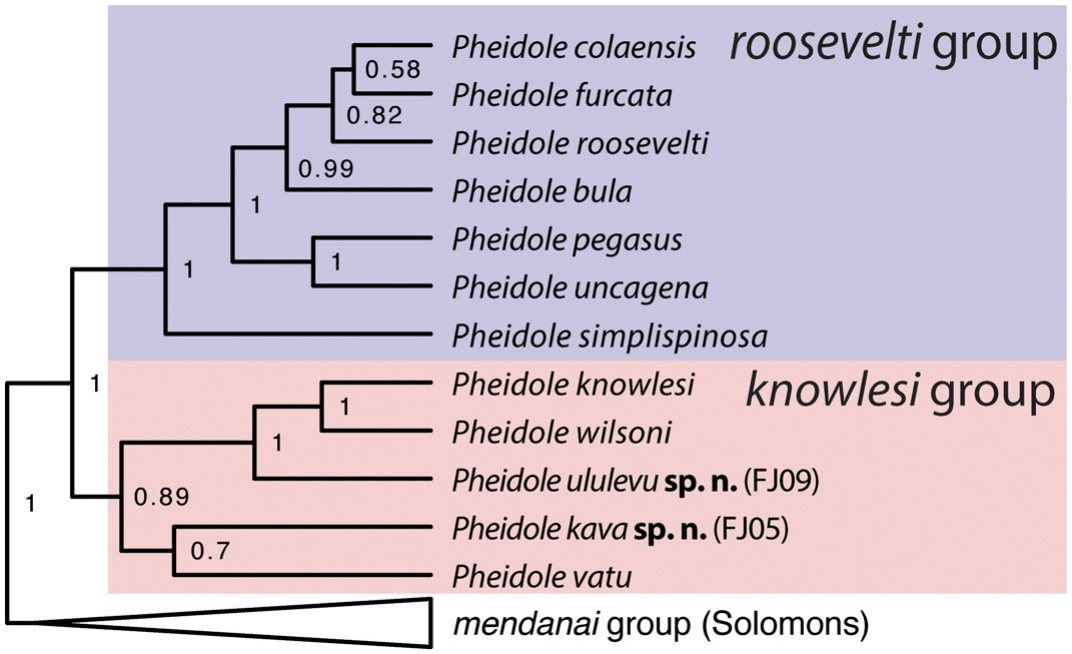
Phylogeny of the Fijian *roosevelti* and *knowlesi* groups and their sister clade. Redrawn from the larger phylogeny in Economo et al. 2015b [26]. One member of the *knowlesi* group, *P*. *caldwelli*, is not represented due to lack of molecular data.

The remaining Fijian *Pheidole* are sister to Solomon Island endemics and nested within a clade that originated in Southeast Asia before spreading to Oceania [25]. The ancestor of the Fijian clade colonized the archipelago during the Miocene ca. 17–10 Ma before radiating into 12 extant species [23]. These species are divided into two clades: the *roosevelti* group, which is notable for its extraordinary spinescence, and the *knowlesi* group, which retains the ancestral non-spinescent *Pheidole* morphology typical of the genus worldwide (Fig 1). The transition into this spinescent form in Fiji was associated with ecological changes and increased geographic restriction, in accordance with the taxon cycle hypothesis [23,24]. The *roosevelti* group was previously revised by Sarnat [22].

The goal of the current contribution is to revise the *knowlesi* group and complete the taxonomic treatment of known *Pheidole* endemic to the Fiji Islands. We describe two new species of the *knowlesi* group, *P*. *kava* sp. n. and *P*. *ululevu* sp. n., and re-describe four species, *P*. *caldwelli* Mann, *P*. *knowlesi* Mann, *P*. *vatu* Mann, and *P*. *wilsoni* Mann.

**Fig 2:**
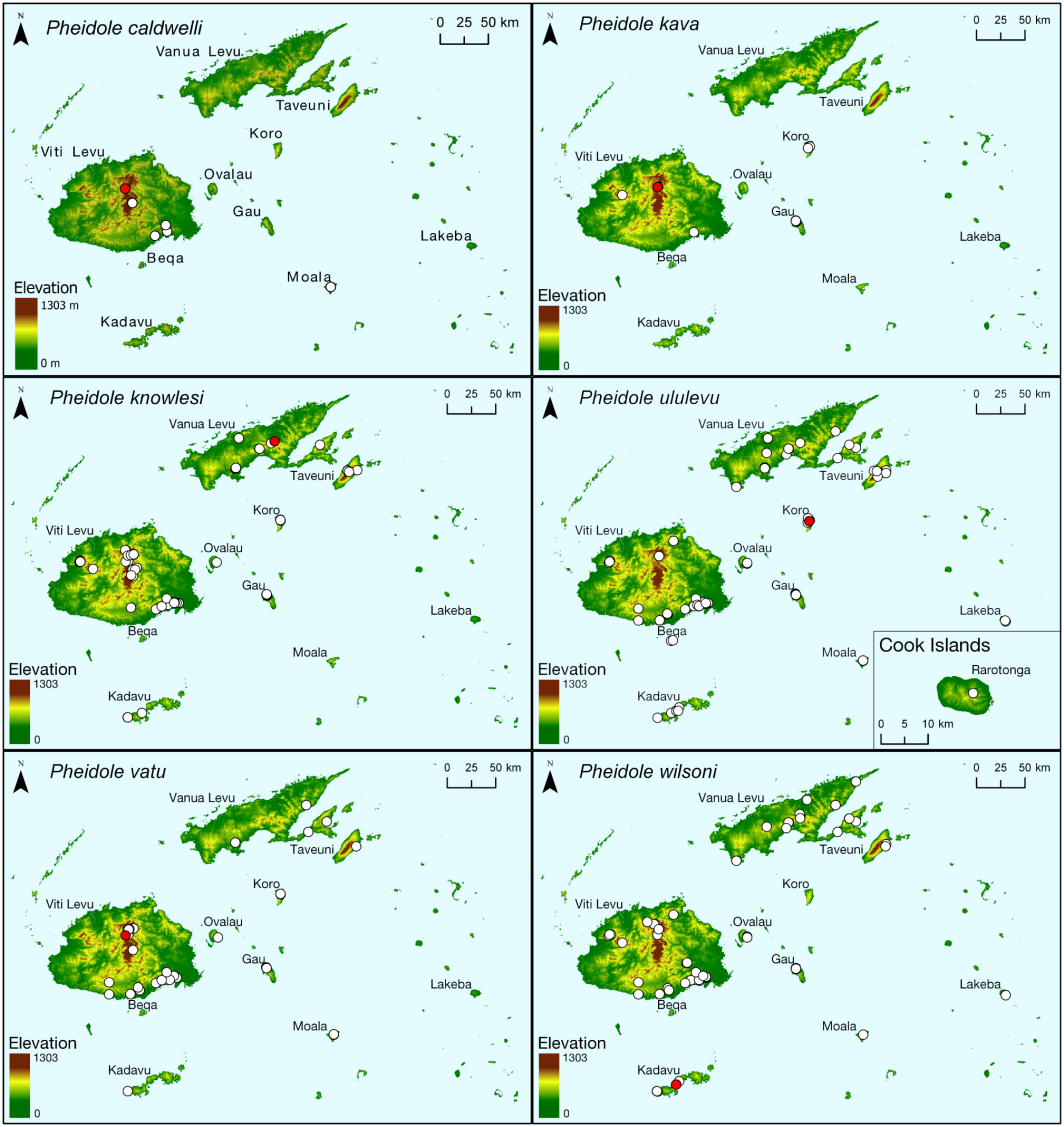
Distribution maps for all species revised in this publication. Type localities are marked in red.

The *knowlesi* group consists of five relatively small-bodied, inconspicuous *Pheidole* species and the slightly larger *Pheidole caldwelli* Mann. The latter seems to be a relatively rare species within this group, and is only recorded from five localities on Viti Levu, and on Moala Island (distribution maps in Fig 2). The species with the widest distribution in this group is *P*. *ululevu* sp. n. It was collected on all sampled Fijian islands and, in addition, on Rarotonga (Cook Islands). The discovery represents the first scientific evidence that Fiji acts not only as a recipient of Pacific island ant diversity from western Melanesia and Indoaustralia, but also as a source of dispersing lineages for the Polynesian archipelagoes to the east. Although five other native Fijian ant species are known from only one other Pacific archipelago, which hints at faunal exchange among remote archipelagoes (Table 1), the case of *P*. *ululevu* is the first confirmed outward dispersal of a species from an endemic Fijian radiation.

**Table 1:** Native Fijian ant species known from only one other Pacific island.

Species	Putative sister population
*Acropyga lauta* Mann	Solomon Is.
*Adelomyrmex hirsutus* Mann	New Caledonia
*Adelomyrmex samoanus* Wilson & Taylor	Samoa
*Pheidole ululevu* Fischer, Sarnat & Economo	Cook Islands
*Strumigenys mailei* Wilson & Taylor	Samoa
*Vollenhovia denticulata* Emery	New Caledonia

In this revision we provide detailed descriptions for both worker subcastes and the queens, together with high-resolution images, distribution maps and identification keys for all six species.

To enhance description and identification efforts, we pursued X-ray microtomography of queens, major workers and minor workers. The presented research is among the first studies in ant taxonomy to utilize micro-CT technology. 3D X-ray scan images, used together with traditional images from multi-focus light-microscopy, illustrate the morphology of specimens more immediately and quantifiably than two-dimensional images alone. For example, exact measurements can be obtained from the 3D pdfs as well as the original scan files without the need of cumbersome loans or museum visits for examining the actual type material. In this respect we are applying a new technique to ant taxonomic research, which has recently gained some momentum in species descriptions of other arthropod groups such as Myriapoda [28–30], polychaete worms [31] and earthworms [32]. Authors of other groups are increasingly using the technique to illuminate the functional morphology and other aspects of study subjects via three-dimensional reconstructions of inner and outer anatomy [33–35]. In both approaches, taxonomy and functional morphology, non-invasive, virtual dissections are an immensely useful tool, enabling researchers to extract new characters and examine different hypotheses without the need of risky and time-consuming specimen shipping, handling, and examination, provided that the original scanning data are stored in open access online repositories.

## Methods

### Museum abbreviations

BPBM Bernice Pauahi Bishop Museum (Honolulu, HI, USA)
MCZC Museum of Comparative Zoology, Harvard, Cambridge (Boston, MA, USA)
USNM United States National Museum of Natural History (Washington D.C., USA)

### Study specimens

**Fig 3:**
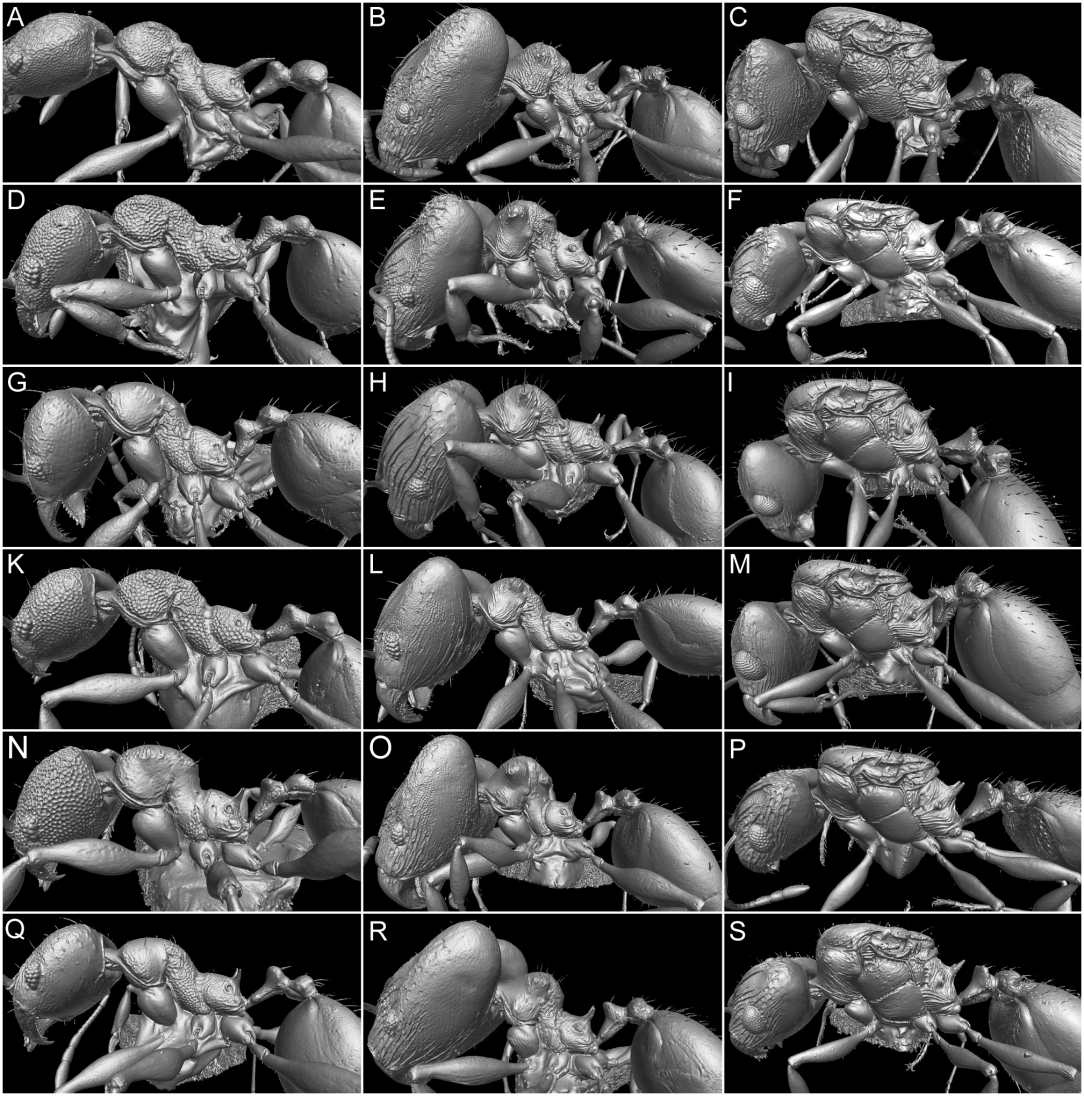
Shaded surface display volume renderings extracted from 3D-scans with specimens seen in profile. *Pheidole caldwelli*: (A) minor worker (CASENT0709600), (B) major worker (CASENT0709599), (C) queen (CASENT0185623). *P*. *kava* sp. n.: (D) minor worker (CASENT0183982), (E) major worker (CASENT0185437), (F) queen (CASENT0194645). *P*. *knowlesi*: (G) minor worker (CASENT0184016), (H) major worker (CASENT0183992), (I) queen (CASENT0184086). *P*. *ululevu* sp. n.: (K) minor worker (CASENT0183250), (L) major worker (CASENT0183915), (M) queen (CASENT0185564). *P*. *vatu*: (N) minor worker (CASENT0185991), (O) major worker (CASENT0184486), (P) queen (CASENT0185452). *P*. *wilsoni*: (Q) minor worker (CASENT0184378), (R) major worker (CASENT0184311), (S) queen (CASENT0184003).

All of the material examined in this study was collected from 2002–2007, including specimens collected as part of the Fiji Terrestrial Arthropod Survey [2,7] and in other collections made by the authors. The ant specimens were collected and stored in ethanol before they were dry-mounted on paper tips and insect pins and are currently deposited in the ant collection in OIST, Okinawa, Japan. The holotype material designated here will be deposited in the BPBM. Paratypes will also be deposited in MCZC and USNM. Data associated with the locality codes listed in the Material Examined sections can be accessed by using the advanced search option in Antweb.org, and are also available in Appendix A of Sarnat & Economo [2]. Additional images along with additional specimen, collection and locality data for all species treated here are available on Antweb.org. Eighteen specimens in total–one queen, one major worker and one minor worker of each species–were selected for micro-CT imaging (Table 2). Profile views of these 18 specimens are presented in Fig 3.

**Table 2:** Specimens used for micro-CT imaging, scan settings and voxel sizes in the resulting scan files.

**Species**	**Sub-/ Caste**	**Specimen Code**	**Voltage (keV)**	**Power (W)**	**Exposure Time (s)**	**Voxel Size (mm)**
*P*. *caldwelli*	major	CASENT0709599	40	3	2	4.22E-03
*P*. *caldwelli*	minor	CASENT0709600	40	3	2	3.49E-03
*P*. *caldwelli*	queen	CASENT0185623	30	2	10	4.57E-03
*P*. *kava*	major	CASENT0185437	45	3	2	3.38E-03
*P*. *kava*	minor	CASENT0183982	45	3	2	2.53E-03
*P*. *kava*	queen	CASENT0194645	55	4	2	3.57E-03
*P*. *ululevu*	major	CASENT0183992	40	3	3	3.18E-03
*P*. *ululevu*	minor	CASENT0184016	50	3.5	2.5	2.16E-03
*P*. *ululevu*	queen	CASENT0184086	40	3	2	3.64E-03
*P*. *knowlesi*	major	CASENT0183915	40	3	3	3.69E-03
*P*. *knowlesi*	minor	CASENT0183250	45	3	3	2.53E-03
*P*. *knowlesi*	queen	CASENT0185564	75	5	2	5.41E-03
*P*. *vatu*	major	CASENT0184486	45	3	2	3.20E-03
*P*. *vatu*	minor	CASENT0185591	45	3	4	2.25E-03
*P*. *vatu*	queen	CASENT0185452	50	4	2	3.69E-03
*P*. *wilsoni*	major	CASENT0184219	40	3	2	3.69E-03
*P*. *wilsoni*	minor	CASENT0184311	45	3	3	3.13E-03
*P*. *wilsoni*	queen	CASENT0184003	75	4.5	2	5.20E-03

### Microtomography

X-ray microtomography (micro-CT/μCT) scans were created with a ZEISS Xradia 510 Versa and the ZEISS Scout and Scan Control System software. The scanned specimens were left attached to their paper point, which was glued to one end of a length (~4cm) of MicroLumen high performance medical tubing (polyimide tubing). Scan settings were selected according to yield optimum scan quality: 4x objective, exposure times between 2 and 10 seconds (the queen of *P*. *caldwelli* was the longest, which previously had been used for non-destructive DNA extraction), binning of two by two pixels, source filter “Air”, voltage between 30 and 75 keV, power between 2 and 5 W, and field mode “normal”. The combination of voltage, power and exposure time was set to yield intensity levels of between 10000 and 15000 across the whole specimen. Scan times varied from 1.5 to 3 hours, depending on exposure times. Full 360 degree rotations were done with a number of 1601 projections. The resulting scans have resolutions of 1013x992x993 (HxWxD) pixels and voxel sizes range between 2.16μm and 5.41μm.

3D reconstruction of the resulting scans was done with XMReconstructor and saved in DICOM file format (default settings; USHORT(16 bit) output data type). These were accessed with Amira software version 6. For rotation videos and still images the *Volren* function was used to create the 3D rendering, the color space range adjusted to minimum so that ant specimens’ exterior surface and pilosity only remained visible (shading: user-defined > coefficients ka = 0.15, kd = 0.4, ks = 0.4; light angle: user-defined > light angle yaw = 0.0357, light angle pitch = –0.1726). The rotation videos were created with *Camera Path* object (7 keyframes, *const*. *velocity* for constant rotation speed) and *movie maker* function (parameters: mpeg format, AntiAlias2, total of 600 frames at 24 frames per second, and resolution of 720 by 480 pixels).

The first step to creating 3D pdfs was to make 3D renderings of ant specimens in Amira using the *Isosurface* function (deselect *compactify*) for exporting surface meshes in the STL file format. These were imported into Meshlab where the number of vertexes per specimen was reduced in three steps to decrease total file size and before importing into Adobe Acrobat. First, the scan files were cleaned from isolated vertexes (Filters > Cleaning and Repairing > Remove isolated pieces (wrt diameter) [set max diameter: 0.05–1%]) and the paper tips on which the ants are mounted were removed. The next step removed all internal vertexes so that only the exoskeleton and the hairs remained (1. Filters > Color Creation and Processing > Ambient Occlusion Per Vertex; 2. Filters > Selection > Select Faces By Vertex Quality (min = 0, max = 0.001); 3. Remove Selected Faces). In the last step, the number of total vertexes was reduced to the final number of <1,000,000 (Filters > Remeshing, Simplification and Reconstruction > Quadratic Edge Collapse Decimation) in order to get a manageable resolution resulting in 3D pdf files less than 40MB in size. They were annotated and exported as 3D pdfs in Adobe Acrobat Pro DC (Version 2015.006.30119) using the Tetra4D Converter plug-in (Version 5.1.2). The 3D pdf and rotation video files are downloadable from the supporting information (Overview Table 3) and the original volumetric datasets (dicom files) have been archived at the Dryad Data Repository (http://datadryad.org, 10.5061/dryad.7d3v4).

**Table 3:** Overview of 3D pdf files and rotation videos available in the supporting information.

File name	Species	Sub-/Caste	Specimen Code	File Type
S1 Fig	*P*. *caldwelli*	major	CASENT0709599	3D PDF
S2 Fig	*P*. *caldwelli*	minor	CASENT0709600	3D PDF
S3 Fig	*P*. *caldwelli*	queen	CASENT0185623	3D PDF
S4 Fig	*P*. *kava*	major	CASENT0185437	3D PDF
S5 Fig	*P*. *kava*	minor	CASENT0183982	3D PDF
S6 Fig	*P*. *kava*	queen	CASENT0194645	3D PDF
S7 Fig	*P*. *knowlesi*	major	CASENT0183992	3D PDF
S8 Fig	*P*. *knowlesi*	minor	CASENT0184016	3D PDF
S9 Fig	*P*. *knowlesi*	queen	CASENT0184086	3D PDF
S10 Fig	*P*. *ululevu*	major	CASENT0183915	3d PDF
S11 Fig	*P*. *ululevu*	minor	CASENT0183250	3D PDF
S12 Fig	*P*. *ululevu*	queen	CASENT0185564	3D PDF
S13 Fig	*P*. *vatu*	major	CASENT0184486	3D PDF
S14 Fig	*P*. *vatu*	minor	CASENT0185591	3D PDF
S15 Fig	*P*. *vatu*	queen	CASENT0185452	3D PDF
S16 Fig	*P*. *wilsoni*	major	CASENT0184311	3D PDF
S17 Fig	*P*. *wilsoni*	minor	CASENT0184378	3D PDF
S18 Fig	*P*. *wilsoni*	queen	CASENT0184003	3D PDF
S1 Vid	*P*. *caldwelli*	major	CASENT0709599	3D rotation video
S2 Vid	*P*. *caldwelli*	minor	CASENT0709600	3D rotation video
S3 Vid	*P*. *caldwelli*	queen	CASENT0185623	3D rotation video
S4 Vid	*P*. *kava*	major	CASENT0185437	3D rotation video
S5 Vid	*P*. *kava*	minor	CASENT0183982	3D rotation video
S6 Vid	*P*. *kava*	queen	CASENT0194645	3D rotation video
S7 Vid	*P*. *knowlesi*	major	CASENT0183992	3D rotation video
S8 Vid	*P*. *knowlesi*	minor	CASENT0184016	3D rotation video
S9 Vid	*P*. *knowlesi*	queen	CASENT0184086	3D rotation video
S10 Vid	*P*. *ululevu*	major	CASENT0183915	3D rotation video
S11 Vid	*P*. *ululevu*	minor	CASENT0183250	3D rotation video
S12 Vid	*P*. *ululevu*	queen	CASENT0185564	3D rotation video
S13 Vid	*P*. *vatu*	major	CASENT0184486	3D rotation video
S14 Vid	*P*. *vatu*	minor	CASENT0185591	3D rotation video
S15 Vid	*P*. *vatu*	queen	CASENT0185452	3D rotation video
S16 Vid	*P*. *wilsoni*	major	CASENT0184311	3D rotation video
S17 Vid	*P*. *wilsoni*	minor	CASENT0184378	3D rotation video
S18 Vid	*P*. *wilsoni*	queen	CASENT0184003	3D rotation video

### Measurements

**Fig 4:**
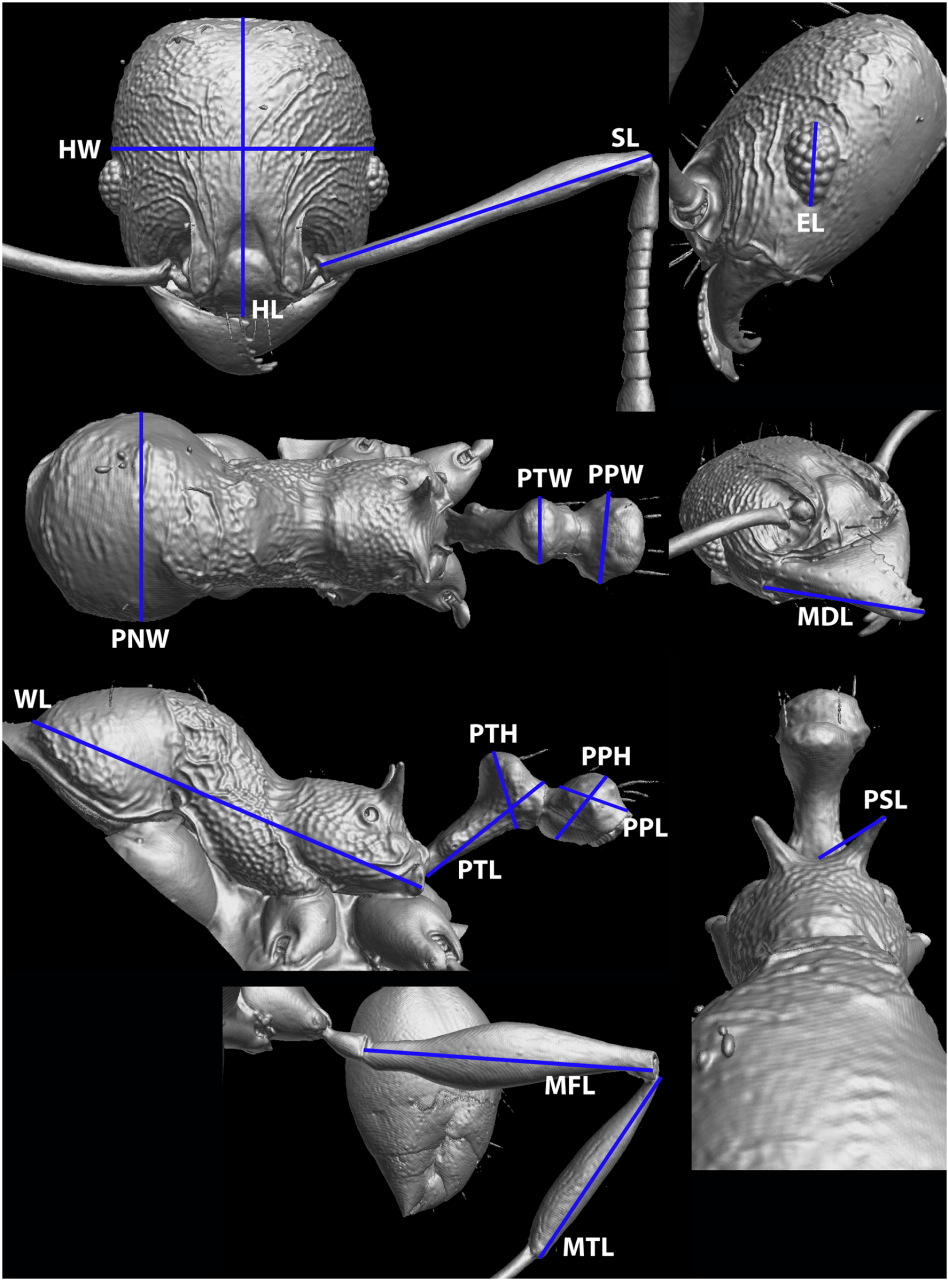
Standard measurements used in this revision. HW–head width, HL–head length, SL–scape length, EL–eye length, MDL–mandible length, PNW–pronotum width, PTW–petiole width, PPW–postpetiole width, WL–Weber’s length, PTL–petiole length, PTH–petiole height, PPL–postpetiole length, PPH–postpetiole height, PSL–propodeal spine length, MFL–metafemur length, MTL–metatibia length.

The following measurements are illustrated in Fig 4 and are the same as in Fischer and Fisher [36]:

HL *head length*: maximum distance from midpoint of anterior clypeal margin to midpoint of posterior margin of head, measured in full-face view; in majors, measured from midpoint of tangent between anterior-most position of clypeus to midpoint of tangent between posterior-most head margin of posterolateral lobes.
HW *head width*: measured at widest point of head, in full-face view behind eye level.
SL *scape length*: maximum scape length, excluding basal condyle and neck.
EL *eye length*: maximum diameter of compound eye measured in oblique lateral view.
MFL *metafemur length*: measured from junction with trochanter to junction with tibia.
MTL *metatibia length*: measured from junction with femur to junction with first tarsal segment.
MDL *mandible length*: maximum length, measured in oblique frontolateral view, from apex to lateral base.
PNW *pronotal width*: maximum width of pronotum measured in dorsal view.
WL *Weber’s length*: diagonal length of mesosoma in profile from anterior point of pronotal slope and excluding neck, to posteroventral margin of propodeum.
PSL *propodeal spine length*: in dorsocaudal view, with apex of measured spine, its base, and center of propodeal concavity between both spines in focus: measurement is taken from apex to base along one axis of a dual-axis micrometer, which is aligned along length of spine, while second axis crosses base of measured spine, and connects base with center of propodeal concavity.
PTL *petiole length*: maximum diagonal length of petiole, measured in profile, from most anteroventral point of peduncle, at or below propodeal lobe, to most posterodorsal point at junction to first helcial tergite.
PTH *petiolar node height*: maximum height of petiolar node measured in lateral view from highest (median) point of node, orthogonally to ventral outline of node.
PTW *petiolar node width*: maximum petiolar node width, measured in dorsal view.
PPL *postpetiole length*: maximum length of postpetiole, measured in profile, from anterior beginning of dorsal slope to posterior juncture of postpetiole and second helcial tergite.
PPH *postpetiole height*: maximum height of postpetiole, measured in profile, from the highest (median) point of node to lowest point of ventral face, often in an oblique line.
PPW *postpetiole width*: maximum width of postpetiole, measured in dorsal view.

### Indices

CI *cephalic index*: HW / HL × 100
SI *scape index*: SL / HW × 100
MDI *mandible index*: MDL / HW × 100
EI *eye index*: EL / HW × 100
FI *metafemur index*: MFL / HW × 100.
PSLI *propodeal spine index*: PSL / HW × 100
LPpI *lateral postpetiole index*: PPL / PPH × 100
DPpI *dorsal postpetiole index*: PPW / PPL × 100
PpWI *postpetiole width index*: PPW / PTW × 100
PpHI *postpetiole height index*: PPH / PTH × 100

### Nomenclatural acts

The electronic edition of this article conforms to the requirements of the amended International Code of Zoological Nomenclature, and hence the new names contained herein are available under that Code from the electronic edition of this article. This published work and the nomenclatural acts it contains have been registered in ZooBank, the online registration system for the ICZN. The ZooBank LSIDs (Life Science Identifiers) can be resolved and the associated information viewed through any standard web browser by appending the LSID to the prefix “http://zoobank.org/”. The LSID for this publication is: urn:lsid:zoobank.org:pub:F076D2FF-EB11-4C1B-8A86-3BC39784DBFC. The electronic edition of this work was published in a journal with an ISSN, and has been archived and is available from the following digital repositories: PubMed Central, LOCKSS.

## Results

### Species synopsis

*P*. *caldwelli* Mann, 1921

*P*. *kava* **sp. n.** Fischer, Sarnat & Economo

*P*. *knowlesi* Mann, 1921

*P*. *ululevu* **sp. n.** Fischer, Sarnat & Economo

*P*. *vatu* Mann, 1921

*P*. *wilsoni* Mann, 1921

### *Pheidole knowlesi* group description

**Fig 5:**
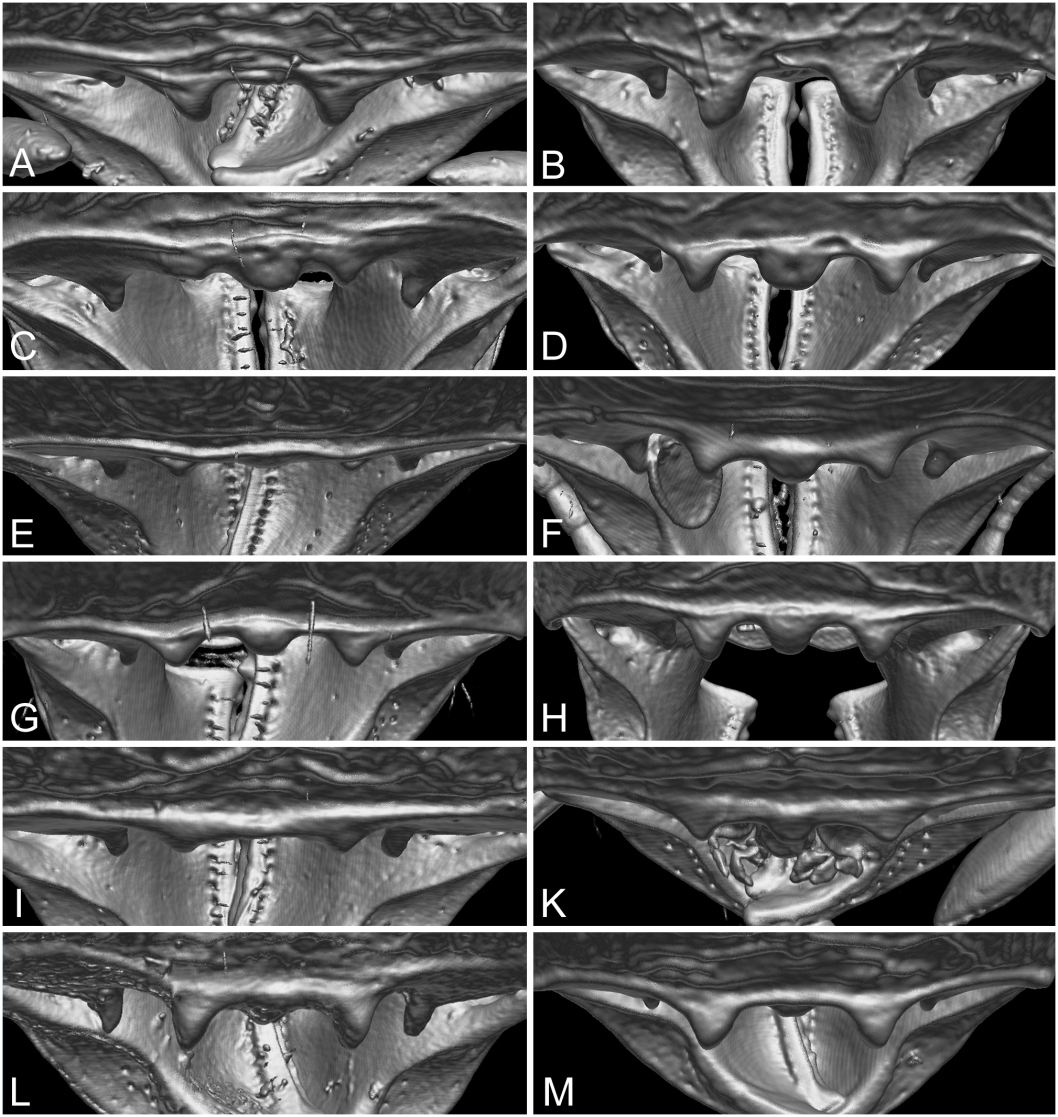
View of major worker and queen hypostomal margin, extracted from 3D-scans as seen from posterior view. *Pheidole caldwelli*: (A) major worker (CASENT0709599), (B) queen (CASENT0185623). *Pheidole kava* sp. n.: (C) major worker (CASENT0185437), (D) queen (CASENT0194645). *Pheidole knowlesi*: (E) major worker (CASENT0183992), (F) queen (CASENT0184086). *Pheidole ululevu* sp. n.: (G) major worker (CASENT0183915), (H) queen (CASENT0185564). *Pheidole vatu*: (I) major worker (CASENT0184486), (K) queen (CASENT0185452). *Pheidole wilsoni*: (L) major worker (CASENT0184311), (M) queen (CASENT0184003).

All members of the *knowlesi* group share the following characteristics:

**Major workers:** head subrectangulate to almost square and slightly longer than wide (CI 88–98), with varying sculpture patterns, from mostly smooth or micropunctate ground sculpture between and posterior of longitudinal rugae on frons and sides to mostly punctate ground sculpture with longitudinal rugae on frons and rugoreticulum posteriorly on posterolateral lobes. Mandibles smooth, with two apical teeth and with usually two small basal teeth on masticatory margin, but *P*. *caldwelli* with only one slightly larger basal tooth. Frontal carinae posteriorly diverging and relatively long, extending almost 2/3 towards posterior head corners, antennal scrobe present and well to moderately well defined. Hypostoma with small to inconspicuous median tooth, flanked by small to relatively large submedian teeth (see Fig 5). Mesosoma in profile rounded or with posterior process on promesonotal declivity. Propodeum armed with well-developed spines, which are slightly shorter than distance between their bases or longer in *P*. *caldwelli*. Postpetiole in profile usually higher than long, in dorsal view on average twice as wide as petiole (PpWI 144–247) and with reduced to moderately long lateral processes.

**Minor workers:** head slightly longer than wide (CI 86–96), weakly oval with convex sides and posterior margin moderately narrow and rounded in *P*. *caldwelli* and some specimens of *P*. *wilsoni* and *P*. *ululevu*, but usually broad and straight to weakly concave medially. Posterior promesonotal process absent in most species, but present in *P*. *caldwelli*. Propodeum in profile about as high as long, spines usually well-developed and spinose, shorter than distance between their bases in most species, but often longer in *P*. *caldwelli*. Petiole with moderately long peduncle, postpetiole shorter than petiole, and about as high as long in profile. Sculpture variable, from partly smooth and shiny or superficially punctate and partly punctate or weakly rugulose to uniformly punctate on almost all surfaces except majority of gaster.

**Queens:** general morphology as in other congeneric species. Head shape square to subrectangulate, usually slightly wider than long (CI 101–111), sides parallel to subparallel, posterior head margin medially weakly concave. Frons with longitudinal rugae, frontal carina relatively long, reaching about 3/4 to 4/5 towards posterior head margin, antennal scrobe about as well defined as in major workers. Mandibles smooth with two apical and usually two small basal teeth along masticatory margin, in *P*. *caldwelli* however only one slightly larger basal tooth present. With antennae in repose, apex of scape not reaching posterior head margin and not surpassing posterior end of antennal scrobe. Hypostomal median tooth from relatively large to absent or inconspicuous, submedian teeth varying from large and stout to small and reduced (see Fig 5). Mesosoma with flight sclerites well-developed, in profile dorsal outline flattened with scutum and scutellum forming one horizontal to weakly convex (in *P*. *knowlesi*) line, scutum with several longitudinal carinae, in *P*. *caldwelli* subparallel and covering entire surface, in other species moderately short to long and forming a V-shape, with remainder of surface smooth and shiny. Postpetiole usually more than two times wider than long (DPpI 206–248) to slightly less than two times wider (DPpI 194) in *P*. *caldwelli*.

### Identification keys for workers and queens of the *knowlesi* group

**Major workers: (modified from Sarnat and Economo 2012 [2])**
1a. Relatively large (HW 1.55–1.62). Propodeal spines longer than distance between their bases (PSLI 23–40). Dorsal surface of petiole and anterior half of first gastral tergite punctate-reticulate. … *P*. *caldwelli*
1b. Considerably smaller (HW 0.73–1.19). Spines slightly shorter than distance between their base (PSLI 14–20). Dorsal surface of postpetiole usually smooth and sculpture on first gastral tergite, if present, limited to anterior quarter or third of segment. … 2
2a. Head posterior to scrobes and on posterolateral lobes smooth and shiny or with weak rugulae but never strongly rugoreticulate. Antennal scrobe smooth to weakly punctate. Head in profile weakly impressed between frons and posterodorsal lobes. … 3
2b. Posterior portion of head strongly rugoreticulate. Antennal scrobe punctate. Head in profile more strongly impressed between frons and posterodorsal lobes.… 5
3a. Posterolateral lobes usually glassy smooth or at most with a few rugulae present, but never punctate. Rugae between eyes and antennal insertions unbranching and lacking reticulation. Antennal scrobe narrow.… *P*. *knowlesi*
3b. Posterolateral lobes with scattered rugulae and often weakly punctate anteriorly and smooth posteriorly, never entirely smooth and shiny. Rugae between eyes and antennal insertions branching and reticulate. Antennal scrobe wider.… 4
4a. Antennal scrobe posteriorly bordered by transverse and arcuate rugulae. Area posterior to antennal scrobe with many branching and reticulated rugulae. Metapleuron mostly smooth and shiny with striae, but not punctate. … *P*. *wilsoni*
4b. Antennal scrobe not posteriorly bordered by transverse and arcuate rugulae. Area posterior to antennal scrobe with mostly longitudinal rugulae that rarely branch or become reticulate. Metapleuron mostly punctate with striae.… *P*. *ululevu*
5a. Gaster with basal third of first tergite striate and punctate. Anterior margin of clypeus notched.… *P*. *vatu*
5b. Gaster mostly smooth, sculptured area reduced to small anterior patch with superficial punctations. Anterior margin of clypeus flat and lacking notch. … *P*. *kava*

**Minor workers: (modified from Sarnat and Economo 2012 [2])**
1a. In profile, promesonotal posterior declivity with distinct process present. Spines longer than distance between their bases (PSLI 27–38). Coxae and legs with weak but distinct punctures present. Postpetiole large and much more voluminous than petiole node in profile. … *P*. *caldwelli*
1b. Promesonotal posterior declivity not bearing distinct process. Spines shorter than distance between their bases (PSLI 15–24). Coxae and legs smooth and shiny, postpetiole in profile not significantly larger than petiole node. … 2
2a. Head dorsum densely and deeply punctate.… 3
2b. Punctures on head dorsum weaker, frons usually only weakly punctate to partly smooth and with weak rugulae present.… 5
3a. Mesosoma largely smooth, at least on lateral pronotum, mesopleuron and promesonotal declivity. In profile promesonotum strongly domed and posterior declivity relatively long and nearly vertical.… *P*. *vatu*
3b. Mesosoma entirely punctate. Promesonotum sloping more gently towards propodeum with short, oblique posterior declivity. … 4
4a. All surfaces of head and mesosoma densely punctate.… *P*. *ululevu*
4b. Head dorsum and mesosoma punctate, but with large smooth area on head venter.… *P*. *kava*
5a. Frons with very superficial punctures and few weak rugulae present. Promesonotal dorsum smooth to superficially punctate and with weak, transverse rugulae present.… *P*. *knowlesi*
5b. Frons weakly punctate without presence of weak rugulae. Promesonotum mostly smooth to superficially punctate and without transverse rugulae. … *P*. *wilsoni*

**Queens:**
1a. Body strongly sculptured. In dorsal view longitudinal rugae subparallel and covering entire length and width of scutum. Postpetiole dorsum with transverse rugulae and anterior third of gaster also coarsely longitudinally rugulose. … *P*. *caldwelli*
1b. Body less sculptured and with several smooth areas. In dorsal view rugae arranged in V-shape and not spreading across entire scutum. Dorsum of postpetiole smooth and gaster never coarsely longitudinally rugulose, at most punctate anteriorly. … 2
2a. Relatively larger species (WL 1.32–1.82) with longer legs (FI 81–88) … 3
2b. Relatively smaller species (WL 1.10–1.36) with shorter legs (FI 69–79) … 5
3a. Posterolateral corners of head with longitudinal rugae present. In anterodorsal view longitudinal rugae on scutum reaching anterior margin. … 4
3b. Posterolateral corners of head without longitudinal rugae present and smooth. In anterodorsal view longitudinal rugae on scutum not reaching anterior margin to strongly reduced. … *P*. *knowlesi*
4a. Hypostoma with median tooth and submedian teeth of unequal size and median tooth distinctly smaller. Dorsal postpetiole and gaster anteriorly rugopunctate. … *P*. *wilsoni*
4b. Hypostoma with median and submedian teeth of equal size. Dorsal postpetiole and gaster anteriorly smooth. … *P*. *ululevu*
5a. Anterior clypeal margin with shallow median notch. Gaster anteroventrally strongly reticulate-punctate. … *P*. *vatu*
5b. Anterior clypeal margin transverse, without median notch. Gaster anteroventrally either smooth or superficially punctate. … *P*. *kava*

### Species accounts

#### *Pheidole caldwelli* Mann

**Fig 6:**
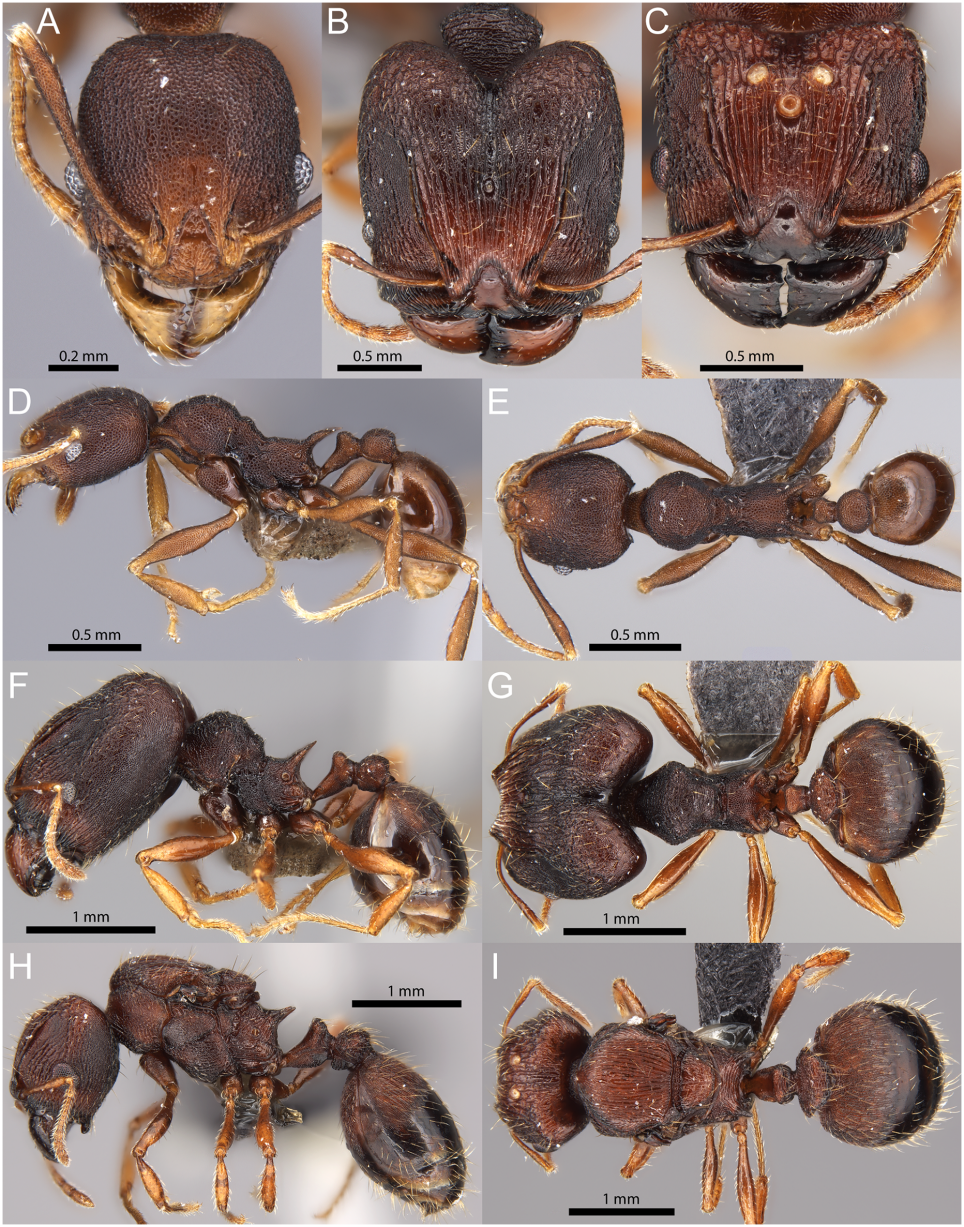
*Pheidole caldwelli* Mann. Minor worker (CASENT0709599): full-face view (A), profile (D), dorsal view (E). Major worker (CASENT0709600): full-face view (B), profile (F), dorsal view (G). Queen (CASENT0185623): full-face view (C), profile (H), dorsal view (I).

(Figs 3A–3C and 6, S1–S3 Figs, S1–S3 Vids)

*Pheidole caldwelli* Mann, 1921 [17]: 434, fig. 14; major worker, minor worker, queen described. Type locality: FIJI, Viti Levu, Nadarivatu (W. M. Mann). Syntypes: 24 minor workers, 4 major workers (MCZC type no. 8697, examined); 11 minor workers, 10 major workers, 3 queens (USNM, examined).

Material examined: **Moala:** Mt. Korolevu 300. **Viti Levu:** Monasavu Dam 800, Korobaba 300, Nabukavesi 300, Waivudawa 300, Nadarivatu 750.

Diagnosis within the *knowlesi* group: **Major worker:** large (HW 1.55–1.62mm); head, mesosoma, postpetiole, most of petiole and anterior half of first gastral tergite entirely sculptured with punctures; frons with irregular, longitudinal rugulae and with median ocellus present, posterolateral lobes rugoreticulate; antennal scrobes relatively narrow; anterior clypeal margin straight with median notch present; hypostoma bearing stout submedian teeth, median tooth absent or inconspicuous. **Minor worker:** head, mesosoma, petiole, postpetiole entirely punctate, often punctures very coarsely and overlain with irregular rugulae. Scapes, dorsum of coxae, legs and anterior 1/3 to 1/4 of first gastral tergite punctate. Promesonotum in profile with short process present on posterior declivity; propodeal spines longer than distance between their relatively massive bases and tapering apically. Postpetiole large and much more voluminous than petiole node in profile. **Queen:** large (WL 1.66), with relatively short legs (FI 75); anterior clypeal margin transverse with median notch; scutum entirely covered with subparallel longitudinal rugulae, anterior half of first gastral tergite strongly longitudinally rugulose and punctate.

**Major workers:** Measurements (n = 5): HW 1.55–1.62 (1.59), HL 1.62–1.66 (1.64), SL 0.79–0.82 (0.81), MDL 0.79–0.81 (0.80), EL 0.18–0.20 (0.19), WL 1.12–1.24 (1.19), PNW 0.69–0.71 (0.70), PTL 0.46–0.49 (0.47), PPL 0.28–0.29 (0.29), PTH 0.29–0.31 (0.30), PPH 0.31–0.35 (0.32), PTW 0.25–0.27 (0.26), PPW 0.46–0.53 (0.50), PSL 0.36–0.38 (0.37), MFL 1.0–1.03 (1.01), MTL 0.73–0.78 (0.75), CI 95–98 (97), SI 50–52 (51), MDI 50–51 (50), EI 12–13 (12), FI 62–66 (64), PpI 67–75 (71), LPpI 83–90 (88), DPpI 164–183 (175), PpWI 170–200 (190), PpHI 103–113 (108). Ground sculpture punctate on head, mesosoma, petiole, postpetiole and anterior half of first gastral tergite and sides of first gastral sternite. Mandibles, median area of clypeus, anterodorsal face of petiole and remainder of gaster smooth and shiny. Head shape in full-face view subrectangulate (CI 95–98) with weakly convex sides. Anterior margin of clypeus transverse, medially notched. In profile, head between frons and posterodorsal lobes with shallow impression. Frontal carinae well-defined anteriorly and medially, extending above antennal scrobe and ending at about 2/3 towards posterior head margin. Frons with ill-defined, irregular rugulae and bearing a single median ocellus in all examined specimens. Irregular, partly reticulate rugulae also present lateral of relatively narrow antennal scrobe, replaced by reticulum posterior of scrobe area and frons. Scape length equals ½ of head width (SI 50–52), when in repose, reaching end of frontal carinae, with sparse, short, and mostly decumbent pilosity and few longer erect hairs along outer edge. Hypostoma without visible median tooth, submedian teeth well-developed and stout. Promesonotum in profile convex with small, rounded posterior process present, metanotal groove not impressed, propodeum compact and short, significantly higher than long in profile, with very short dorsal face and oblique posterior declivity. Spines long, acute and thick at base. In dorsal view, pronotal humeri with very short, but sharply angulate and projecting. Punctuate sculpture on mesosoma mostly covered by weak, irregular rugoreticulum. Coxae often with patches of weak to superficial sculpture. Metatibia pilosity same as scape pilosity. Standing hairs short, suberect, not abundant, of yellow golden colour, shorter, decumbent pilosity present as well. Colour reddish to dark brown, antennae and legs yellowish light brown.

**Minor workers:** Measurements (n = 5): HW 0.55–0.67 (0.63), HL 0.62–0.75 (0.70), SL 0.63–0.75 (0.71), MDL 0.42–0.46 (0.44), EL 0.12–0.15 (0.13), WL 0.80–0.93 (0.88), PNW 0.38–0.45 (0.43), PTL 0.28–0.34 (0.31), PPL 0.18–0.21 (0.20), PTH 0.16–0.18 (0.17), PPH 0.17–0.19 (0.18), PTW 0.13–0.17 (0.15), PPW 0.20–0.25 (0.24), PSL 0.15–0.25 (0.22), MFL 0.72–0.84 (0.79), MTL 0.56–0.63 (0.60), CI 89–92 (90), SI 110–115 (113), MDI 66–80 (71), EI 21–22 (21), FI 124–131 (127), PpI 53–57 (55), LPpI 105–117 (110), DPpI 111–123 (118), PpWI 148–172 (161), PpHI 109–113 (110). Ground sculpture punctate on head including scapes, mesosoma, petiole, postpetiole, dorsum of coxae, legs and anterior third of first gastral tergite. Punctures on head and mesosoma often coarse and overlain by weak, irregular reticulum. Mandibles and remainder of coxae and gaster smooth and shiny. Head shape oval with convex sides, posterior margin moderately wide with rounded corners and medially very shallowly concave. Eyes relatively small, with 6–7 ommatidia in the longest row. Scapes, when in repose, surpassing posterior head margin by about one eye length, scape pilosity very short and erect to suberect. Promesonotal outline in profile convex, broken by a short to distinct process present on posterior declivity, sometimes followed by a second, much smaller process before very shallowly impressed metanotal groove. Propodeum about as high as long, spines often very long, stout and apically acute, at least as long as distance between their bases—up to about twice as long. Metatibia pilosity similar to scape pilosity. Postpetiole large and much more voluminous than petiole, higher than petiole (PpHI 109–113), slightly longer than high (LPpI 105–117), and in dorsal view about 1.2 times wider than long (DPpI 111–123). Standing hairs on head and mesosoma almost absent, only few short erect hairs present on anterior head and posterior gaster. Other areas with shorter erect to suberect pilosity present. Head and body reddish to dark brown, mandibles, funiculus and legs slightly lighter coloured.

**Queen:** Measurements (n = 1): HW 1.33, HL 1.20, SL 0.75, MDL 0.71, EL 0.30, WL 1.66, PNW 1.1, PTL 0.6, PPL 0.35, PTH 0.44, PPH 0.47, PTW 0.39, PPW 0.68, PSL 0.39, MFL 1.0, MTL 0.74, CI 111, SI 56, MDI 53, EI 23, FI 75, PSLI 29, LPpI 74, DPpI 194, PpWI 174, PpHI 107. Head wider than long (CI 111), anterior margin of clypeus with median notch. Scapes relatively short (SI 56), with very short subdecumbent to suberect pilosity and with few longer, erect hairs along outer edge. Head punctate, frontal carinae long and reaching about 4/5 towards posterior head margin. Frons with long, uninterrupted longitudinal rugae, sides and area posterior of ocelli and narrow, but well-developed, scrobes rugoreticulate. Eyes relatively small (EI 23). Median hypostomal tooth reduced or inconspicuous, submedian teeth large and stout. Mesosoma coarsely rugulose-punctate without smooth patches. Scutum with many subparallel carinae covering the whole length and width of the sclerite. Propodeum laterally with longitudinal and oblique rugulae, spines relatively long (PSL 0.39), wide at bases and apex blunt. Petiole strongly convex ventrally, punctate and rugulose, only median area of anterodorsal face smooth. Postpetiole in profile about as high as petiole with convex ventral process, about 1.75 times as wide as petiole (PpWI 174) with laterally extended, angulate corners, its dorsum with transverse rugulae. Standing hairs moderately long and abundant, mostly suberect and yellowish light brown. Colour reddish brown, mandibles and smooth part of gaster darker, antennae and legs slightly lighter colored.

Biogeography & Ecology: About *Pheidole caldwelli*, Mann [17] wrote that it was “*very common in*, *and apparently restricted in distribution to*, *the mountains about Nadarivatu* (Viti Levu), *where numerous colonies were found beneath stones and logs*”. R. W. Taylor collected a series of workers (stored at ANIC) from a rotten log in rainforest also in Nadarivatu district in 1962. In the recent ant survey this species was also collected from localities in the upland forests of southeastern Viti Levu and a lone worker was found on Moala Island. These collections were all made from sifted leaf-litter in primary rainforest and in elevations between 300 and 800m.

Comments: This species is rather distinct within the *knowlesi* group and easily distinguishable from the other five species by its larger size and the extent of punctate and rugose sculpture, which covers almost every surface of the body, including the anterior part of the gaster. The major and minor workers are also characterized by distinctly longer propodeal spines that are at least as long as, to distinctly longer than the distance between their bases and the presence of a process at the promesonotal posterior declivity. Major workers and queens also differ from the other species in the group in having large, stout submedian hypostomal teeth and an absent median tooth. The minors can also be recognized by the relatively large and voluminous postpetiole. *Pheidole caldwelli* has morphological affinities to both the *knowlesi* and *roosevelti* groups, but preliminary next-generation sequencing analysis (unpublished data) places it as part of the monophyletic *knowlesi* group, and thus we include it therein. Shared characters between *P*. *caldwelli* and species from the *roosevelti* group are longer spines, presence of a promesonotal process and its larger size. A unique character state for *P*. *caldwelli* that sets it apart from all other species of the two groups is the complete absence of the median hypostomal tooth in major workers and queens.

#### *Pheidole kava* Fischer, Sarnat and Economo sp. n

**Fig 7:**
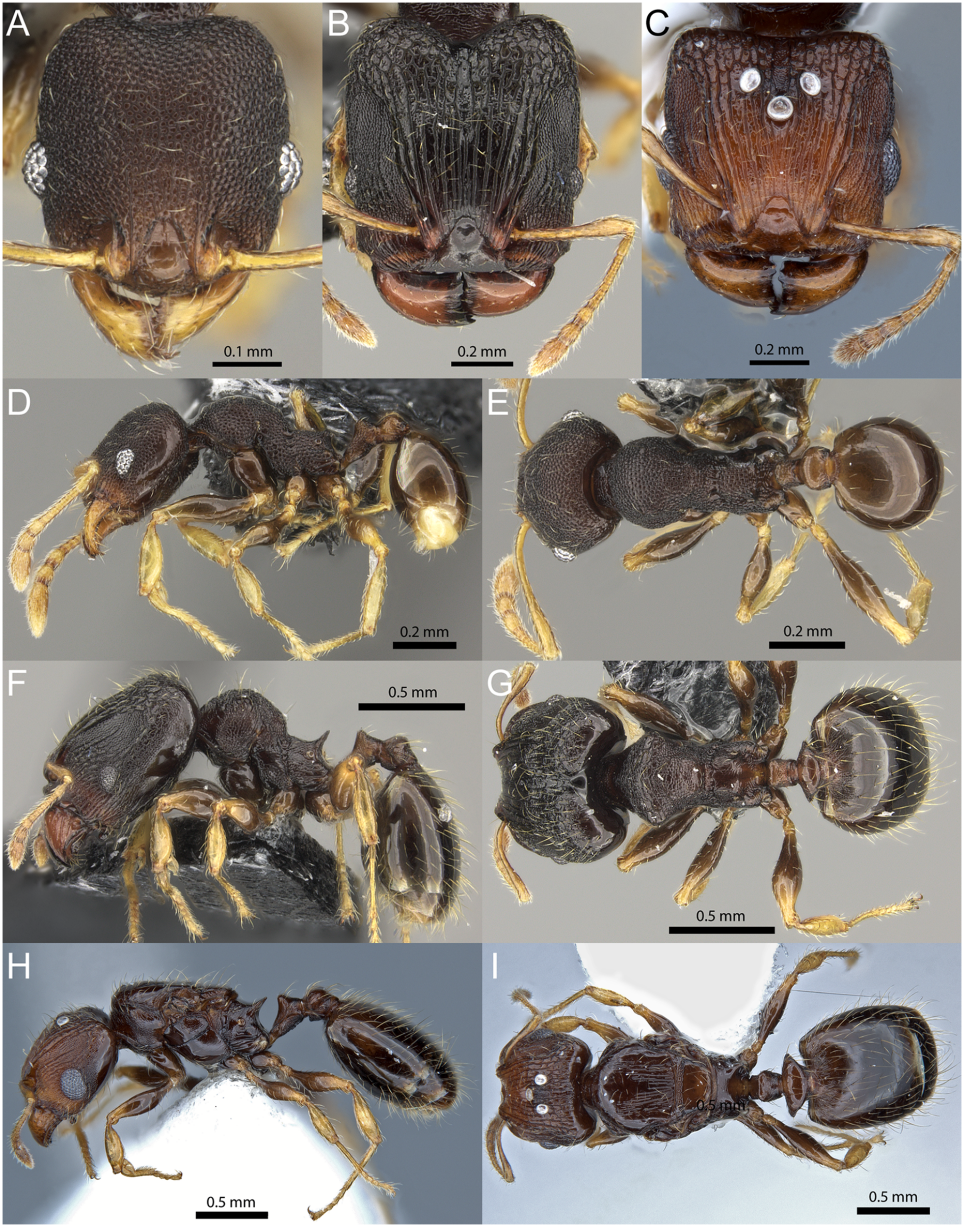
*Pheidole kava* sp. n. Minor worker (paratype, CASENT0183943): full-face view (A), profile (D), dorsal view (E). Major worker (holotype, CASENT0183620): full-face view (B), profile (F), dorsal view (G). Queen (CASENT0194645): full-face view (C), profile (H), dorsal view (I).

(Figs 3D–3F and 7, S4–S6 Figs, S4–S6 Vids) [urn:lsid:zoobank.org:act:3245118C-BAA6-4CF4-97E3-D59DBC055335]

This species was previously treated as morphospecies *P*. FJ05 in the following publications: Sarnat & Economo 2012 [2], Sarnat & Moreau 2011 [23], Economo & Sarnat 2012 [24], Economo et al. 2015a [25] & 2015b [26].

**Holotype:** Major worker (BPBM, specimen code CASENT0183620), FIJI, Viti Levu, Monasavu Rd., 1.75km SE Waimoque Settlement, -17.6703, 177.994, 850m, rainforest/road edge, nesting in ant plant, 28.viii.2006 (*E*.*M*. *Sarnat*).

**Paratypes:** 4 major workers (MCZC, CASENT0183863; USNM, CASENT0183925; CASENT0183626; CASENT0183675), 3 minor workers (BPBM, CASENT0183943; MCZC, CASENT0183979; USNM, CASENT0183982), 1 dealate queen, same data as holotype (CASENT0183961).

Other material examined: **Gau:** Mt. Delaco 408, 475, 490, 505, 575, 625, 675. **Koro:** Mt. Kuitarua 440 b; Nasau 465 a, 470 (4.4 km). **Viti Levu:** Naikorokoro 300; Vaturu Dam 575 b; Waimoque 850.

Diagnosis within the *knowlesi* group: **Major worker:** small (WL 0.85–0.97mm); head heavily sculptured with coarsely rugoreticulate posterolateral lobes, broad and punctate antennal scrobes; anterior clypeal margin straight without median notch; in profile, head impressed between frons and posterior head margin and lobes; gaster smooth, except for superficially punctate spot anteriorly near postpetiole. **Minor worker:** promesonotal profile low with relatively short, oblique mesonotal declivity; sculpture on head and body uniformly punctate, except for smooth head venter, postpetiole dorsum, and gaster. **Queen:** small (WL 1.12–1.36mm), with short legs (FI 69–72); anterior clypeal margin transverse without median notch; head sculpture between longitudinal rugae punctate, rugae near posterior head margin reticulate; gaster smooth, sometimes with weakly rugopunctate area anterodorsally near postpetiole.

**Major workers:** Measurements (n = 7): HW 0.94–1.06 (0.99), HL 1.00–1.10 (1.05), SL 0.46–0.52 (0.50), MDL 0.45–0.57 (0.53), EL 0.15–0.18 (0.16), WL 0.85–0.97 (0.92), PNW 0.56–0.65 (0.59), PTL 0.34–0.38 (0.36), PPL 0.18–0.21 (0.19), PTH 0.21–0.25 (0.22), PPH 0.22–0.25 (0.23), PTW 0.18–0.20 (0.19), PPW 0.35–0.45 (0.40), PSL 0.17–0.20 (0.19), MFL 0.56–0.66 (0.62), MTL 0.43–0.48 (0.46), CI 92–96 (94), SI 48–53 (51), MDI 46–59 (53), EI 15–18 (16), FI 58–66 (63), PpI 61–75 (67), LPpI 78–91 (83), DPpI 184–228 (207), PpWI 189–225 (211), PpHI 100–110 (103). Ground sculpture punctate to weakly punctate on head dorsum, mesosoma, lateroventral petiole and postpetiole. Spaces between rugae on frons and posterolateral lobes, on propodeal dorsum and posterior declivity, and on anterodorsal gaster superficially punctate. Mandibles, clypeus, most of head venter, dorsal waist segments and majority of gaster smooth and shiny. Head rectangular, sides parallel, posteriorly about as wide as anteriorly or slightly narrower. Anterior margin of clypeus transverse to weakly impressed medially. Head in profile slightly impressed between frons and posterodorsal lobes. Frons slightly convex in profile view and in full-face view slightly elevated compared to sides and posterolateral lobes, with well-defined longitudinal rugae, rarely with a single median ocellus present. Posteriorly, rugae becoming weakly reticulate, posterolateral lobes coarsely rugoreticulate. Sides of head punctate with weakly rugoreticulate parts. Scrobe area punctate, well-defined by long, laterally-curving frontal carinae and relatively strongly depressed posteriorly towards sides of the head. Carinae reaching to posterior end of antennal scapes when in repose, but ending well before posterior lobes. Scape length about ½ of head width (SI 48–53), almost reaching posterior 1/3 of head, with short, mostly decumbent pilosity and few longer erect hairs along outer edge. Hypostomal teeth variable, usually with a pronounced and wide median tooth and two shorter submedian teeth (Viti Levu), but sometimes only two submedian and median tooth almost absent, or with a very big median tooth and two very reduced submedian teeth (Gau). Promesonotum in profile convex without posterior process, obtusely angulate towards steep posterior declivity. Metanotal groove not impressed, propodeum short and compact, significantly higher than long in profile, with oblique dorsal face, declining towards moderately long and slender spines. In dorsal view, pronotal humeri weakly projecting. Mesosoma punctuate and transversely rugoreticulate on katepisternum and dorsopropodeum, and sometimes on lateral pronotum and propodeum (Gau, Koro), with superficial sculpture or smooth. Metatibia pilosity mostly fine, decumbent with very few suberect hairs along outer edge. Petiole and postpetiole laterally and ventrally weakly punctate, dorsomedially smooth. Gaster smooth and shiny with weakly to superficially punctate area anteriorly near postpetiole. Standing hairs suberect, relatively flexuous and abundant, on gaster longer and with a relatively thicker base, shorter pilosity decumbent.

**Minor workers:** Measurements (n = 7): HW 0.37–0.45 (0.41), HL 0.40–0.48 (0.44), SL 0.34–0.43 (0.40), MDL 0.23–0.29 (0.26), EL 0.09–0.11 (0.10), WL 0.45–0.56 (0.51), PNW 0.25–0.30 (0.28), PTL 0.16–0.24 (0.20), PPL 0.08–0.10 (0.09), PTH 0.09–0.12 (0.10), PPH 0.08–0.11 (0.10), PTW 0.07–0.09 (0.08), PPW 0.12–0.16 (0.14), PSL 0.08–0.10 (0.09), MFL 0.33–0.41 (0.37), MTL 0.24–0.30 (0.27), CI 91–96 (93), SI 93–100 (96), MDI 61–67 (64), EI 24–27 (25), FI 89–92 (90), PpI 47–56 (50), LPpI 87–100 (92), DPpI 142–164 (154), PpWI 150–180 (165), PpHI 89–96 (93). Head dorsum, mesosoma and petiole laterally coarsely punctate. Mandibles, clypeus, majority of head venter, petiole dorsum medially, postpetiole and gaster smooth and shiny. Head subrectangular, sides weakly convex, posterior margin wide and medially slightly concave, posterior corners rounded. Eyes relatively small, with 6 ommatidia in the longest row. Scapes reaching and barely surpassing posterior head margin, scape pilosity decumbent to subdecumbent with few, very short suberect hairs along outer edge. Promesonotal outline in profile compact, convex, with steep but short posterior declivity, metanotal groove very shallow, with barely visible crossribs, propodeum distinctly higher than long, spines relatively short-spinose, acute, distinctly shorter than distance between their bases. Metatibia pilosity mostly decumbent. Postpetiole almost as high as petiole (PpHI 89–96), slightly higher than long (LPpI 87–100), in dorsal view about 1.5 times wider than long (DPpI 142–164). Long standing hairs on head and mesosoma almost absent, with only few hairs on head and on gaster longer than sparse, short and relatively coarse, decumbent to suberect pilosity. Head and body dark brown, mandibles, antennae, tibiae and tarsi yellow.

**Queens:** Measurements (n = 3): HW 0.80–0.96 (0.88), HL 0.78–0.91 (0.84), SL 0.46–0.57 (0.51), MDL 0.46–0.57 (0.51), EL 0.24–0.27 (0.26), WL 1.12–1.36 (1.22), PNW 0.68–0.87 (0.75), PTL 0.41–0.51 (0.45), PPL 0.21–0.31 (0.24), PTH 0.25–0.28 (0.27), PPH 0.25–0.29 (0.27), PTW 0.22–0.27 (0.24), PPW 0.45–0.46 (0.46), PSL 0.19–0.24 (0.21), MFL 0.57–0.75 (0.64), MTL 0.42–0.57 (0.49), CI 102–105 (104), SI 57–59 (58), MDI 56–59 (58), EI 28–30 (29), FI 69–72 (70), PSLI 23–25 (24), LPpI 54–84 (70), DPpI 214–219 (217), PpWI 196–205 (200), PpHI 76–104 (93). Head slightly wider than long (CI 102–105). Anterior margin of clypeus transverse, without distinctly concave median notch. Scapes relatively short (SI 57–59), pilosity mostly decumbent with longer suberect hairs along outer edge. Head dorsum punctate, with longitudinal rugae, the rugae becoming slightly reticulate near posterior head margin. Hypostomal margin with broad, well-developed median tooth and submedian teeth also large, but narrower. Oblique carinae on scutum not reaching anterior margin in anterodorsal view. Mesosoma mostly smooth and shiny, but lateropronotum punctate-rugulose and weak to superficial punctures present at anterior and lateral pronotum, posterior anepisternum, and lateropropodeum, propodeum laterally also with abundant oblique rugulae. Petiole laterally and often dorsally weakly punctate. Postpetiole about twice as wide as petiole (PpWI 196–205), dorsally smooth, its anterior margin in dorsal view convex, lateral corners widely extended. Gaster smooth, sometimes with weakly rugopunctate area anteriorly near postpetiole. Standing hairs moderately long and abundant, suberect and yellow. Color black or dark brown, anterior of head, mandibles, antennae and legs lighter colored.

Biogeography & Ecology: Within the *knowlesi* group, *Pheidole kava* is relatively rarely collected. It was found on only three of the sampled islands (Viti Levu, Gau, and Koro) from elevations ranging betwen 300 and 850m. *Pheidole kava* was collected from leaf-litter, foraging on the ground, from nests inside dead branches, under stones and in ant plants. Its habitat seems to be restricted to rainforest and bryophyte forest.

Comments: The Koro major worker specimens deviate slightly from those of Gau and Viti Levu by a more smooth and shiny lateral mesosoma, a slightly deeper impression between the frons and the posterior margin of head, and a postpetiole that is wider and more smooth and shiny in dorsal view. Major workers of *Pheidole kava* are most similar to those of *P*. *vatu* in head sculpture and size. Workers and queens of the two species tend to be the smallest in the *knowlesi* group. The majors of *P*. *kava* can be easily distinguished by an almost completely punctate versus a mostly smooth and shiny mesosoma in *P*. *vatu* and by absence of a median clypeal notch in *P*. *kava*. The queens of these two species also share several characters, such as the smaller size and the flatter mesosoma shape in profile. In contrast, the minor workers of *P*. *kava* are most similar to those of *P*. *ululevu*. They are almost exclusively distinguishable by the extent of punctures present on the head. While the minors have dense punctures on the head dorsum, but a smooth head venter in *P*. *kava*, the heads are densely punctate dorsally and ventrally in *P*. *ululevu*. In the workers of *P*. *vatu*, the promesonotum is also significantly higher domed than in *P*. *kava* and the other *knowlesi* group species. Interestingly, *Pheidole kava* displays a rather large variability in the expression of the major workers’ hypostomal teeth (see description).

Etymology: This species is named after the Pacific islands’ mildly narcotic drink, which is brewed from the roots of the kava plant (*Piper methysticum*). The name is a noun in apposition and thus invariant.

#### *Pheidole knowlesi* Mann

**Fig 8:**
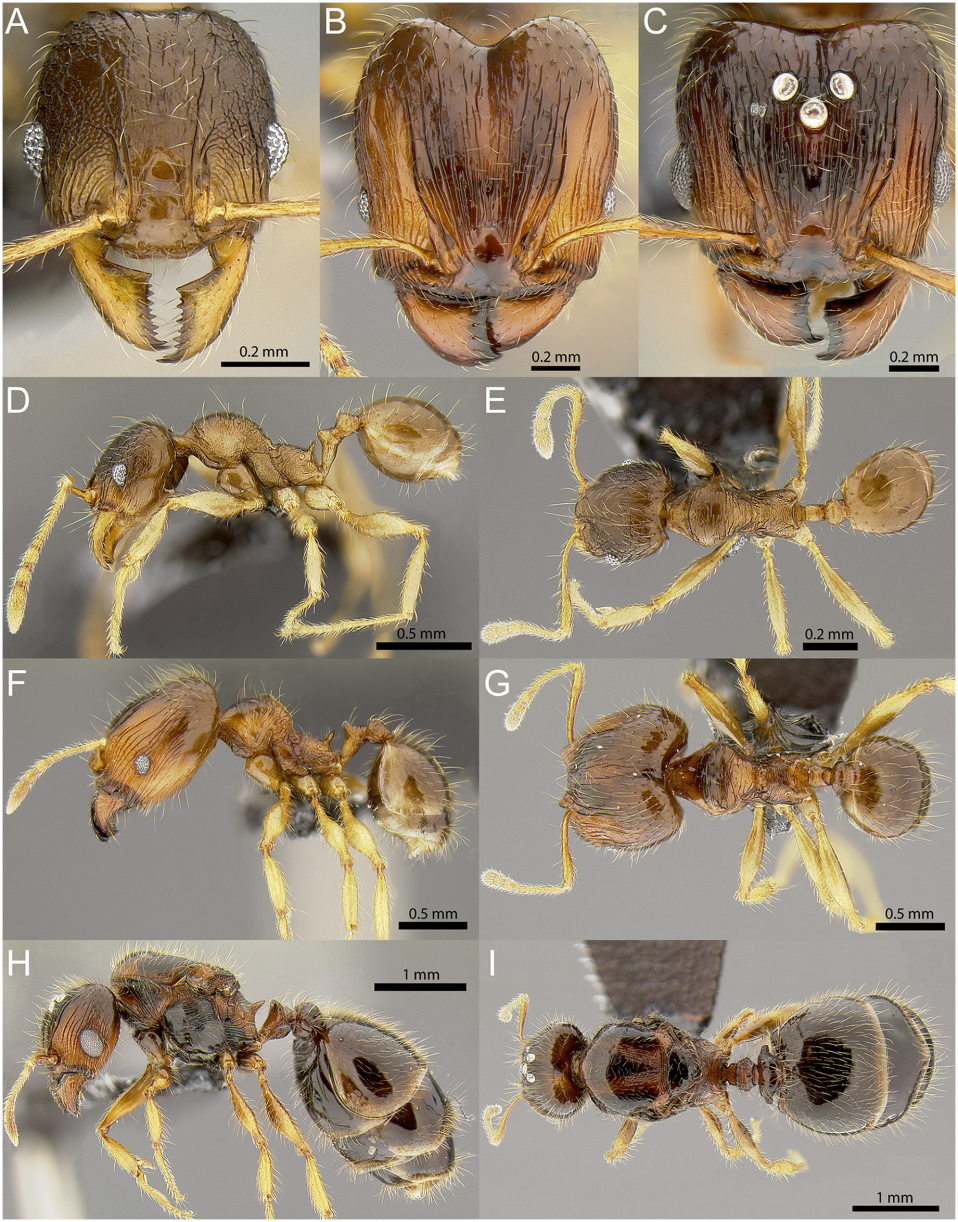
*Pheidole knowlesi* Mann. Minor worker (CASENT0171041): full-face view (A), profile (D), dorsal view (E). Major worker (CASENT0171097): full-face view (B), profile (F), dorsal view (G). Queen (CASENT0171042): full-face view (C), profile (H), dorsal view (I).

(Figs 3G–3I and 8, S7–S9 Figs, S7–S9 Vids)

*Pheidole knowlesi* Mann, 1921 [17]: 436, fig. 13a; FIJI, Vanua Levu, Suene; major worker, minor worker, queen described (MCZC, examined).

*Pheidole knowlesi* subsp. *extensa* Mann, 1921: 438; FIJI, Viti Levu, Nadarivatu; major worker, minor worker described (MCZC, examined). Synonym of *P*. *knowlesi* (Sarnat & Economo 2012) [2].

Material examined: **Gau:** Navukailagi 356, 387, 415, 432, 475, 480, 490, 496, 505, 522, 535, 564, 575, 675, 717. **Kadavu:** Mt. Washington 700, 760, 800; Moanakaka 128; Vanua Ava b. **Koro:** Mt. Kuitarua 440 b, 500; Nasau 465 a. **Ovalau:** Levuka 500. **Taveuni:** Devo Peak 1187; Mt. Devo 734, 775 a, 1064; Tavoro Falls 100; Tavuki 734. **Vanua Levu:** Kilaka 61, 98, 146; Mt. Delaikoro 699, 734; Mt. Vatudiri 570, 641; Rokosalase 118; Suene; Vusasivo Village 342 b. **Viti Levu:** Colo-i-Suva 200, 300, 325, 340 b, 372, 460; Colo-i-Suva Forest Park 220; Korobaba 300; Lami 300, Mt. Tomanivi 700, 700b, 950, 1300; Monasavu 800, 800 a, 850; Monasavu Dam 600, 800, 1000; Mt. Batilamu 840 c; Mt. Evans 800; Mt. Naqaranabuluti 1050; Mt. Rama 300; Nabukavesi 300; Nadarivatu 750; Naikorokoro 300; Nakobalevu 340; Naqaranabuluti 860; Nasoqo 800 a, 800 c, 800 d; Navai 700, 863, 930, 1020; Nuku 50; Savione 750 b, 800; Vaturu Dam 575 b; Waimoque 850; Waivudawa 300.

Diagnosis within the *knowlesi* group: **Major worker:** slightly larger (WL 0.96–1.13mm); head surface shiny with small, superficially punctate spots between rugae on frons and sides, posterolateral lobes smooth; head in profile not or only weakly impressed between frons and posterior head margin and lobes. **Minor worker:** head dorsum medially smooth with superficial punctures, often with irregular rugulae on frons and slightly reticulate sculpture laterally; promesonotum with weak or faint transverse rugulae dorsally, low in profile with gently sloping posterior declivity. **Queen:** large (WL 1.64–1.82) with relatively long legs (FI 85–88); clypeus anteriorly with distinct median notch, space between rugae and near posterior head margin usually smooth and posterolateral corners of head without rugae, rugae on scutum not reaching anterior margin or strongly reduced.

**Major workers:** Measurements (n = 7): HW 0.91–1.16 (1.08), HL 1.01–1.22 (1.13), SL 0.59–0.68 (0.63), MDL 0.57–0.65 (0.61), EL 0.16–0.18 (0.17), WL 0.96–1.13 (1.04), PNW 0.53–0.60 (0.56), PTL 0.35–0.40 (0.37), PPL 0.17–0.19 (0.18), PTH 0.20–0.23 (0.22), PPH 0.18–0.22 (0.20), PTW 0.15–0.17 (0.17), PPW 0.30–0.35 (0.33), PSL 0.16–0.19 (0.17), MFL 0.75–0.88 (0.81), MTL 0.53–0.64 (0.59), CI 90–97 (95), SI 55–67 (59), MDI 53–63 (56), EI 15–18 (16), FI 70–86 (76), PpI 53–61 (58), LPpI 83–100 (90), DPpI 164–194 (181), PpWI 182–212 (196), PpHI 89–102 (93). Posterior head margin, posterolateral lobes, dorsal postpetiole and gaster glassy smooth. Remainder of head and promesonotum usually with superficial punctures between rugae, but sometimes smooth. Propodeum, katepisternum, anepisternum, and lateral petiole from weakly rugopunctate to superficially punctate with some areas appearing smooth. Head subrectangular, sides convex and curved towards posterior head corners; posteriorly about as wide as anteriorly or slightly narrower. Mandibles and clypeus smooth, anterior margin weakly impressed medially. Antennal socket located in excavated pit, frontal lobes translucent. In profile view dorsal head outline simple and convex, without distinct impression between frons and posterolateral lobes. Hypostomal margin with small median tooth and submedian teeth. Frons and sides of head with well-defined longitudinal rugae. Frontal carina sharply defined and raised, bordering whole length of scrobe, reaching posterior end of scapes when in repose and ca. ¾ towards posterior head margin. Antennal scrobe narrow and often very weakly defined. Antennal scape length more than ½ of head width (SI 55–67), slightly surpassing anterior 2/3 of head, with decumbent pilosity and longer erect hairs along outer edge. Promesonotum in profile convex. Posterior declivity ranging from shallowly oblique to weakly angulate. Metanotal groove narrowly impressed. Propodeum in profile as long as high to slightly longer, spines about as long as distance between their bases. In dorsal view pronotal humeri angulate, but not projecting. Promesonotum with transverse rugulae. Metatibia pilosity mostly decumbent or subdecumbent with long suberect hairs along outer edge. Petiole laterally and ventrally weakly punctate, petiolar dorsum and postpetiole smooth and shiny. Postpetiole with acutely angulate lateral processes, on average 2 times wider than petiole (PpWI 182–212) and 1.8 times wider than long (DPpI 167–194). Standing hairs abundant, mostly erect, long and flexuous, with very fine hairs of medium length and shorter decumbent to subdecumbent pilosity. Color ranging from orange to light brown to darker reddish brown, antennae and legs lighter.

**Minor workers:** Measurements (n = 7): HW 0.50–0.58 (0.53), HL 0.55–0.65 (0.59), SL 0.53–0.62 (0.57), MDL 0.34–0.42 (0.37), EL 0.11–0.13 (0.12), WL 0.67–0.83 (0.74), PNW 0.33–0.40 (0.35), PTL 0.19–0.29 (0.23), PPL 0.11–0.13 (0.12), PTH 0.12–0.15 (0.13), PPH 0.10–0.13 (0.11), PTW 0.09–0.11 (0.10), PPW 0.14–0.18 (0.15), PSL 0.08–0.11 (0.09), MFL 0.54–0.68 (0.60), MTL 0.37–0.50 (0.43), CI 89–96 (91), SI 105–110 (107), MDI 64–72 (68), EI 20–25 (22), FI 105–118 (112), PpI 40–49 (43), LPpI 100–115 (103), DPpI 121–143 (130), PpWI 142–162 (151), PpHI 80–89 (84). Majority of head, mesosoma, and lateral petiole weakly to superficially punctate, often overlain with irregular rugulae. Mandibles, clypeus, posterior head margin, dorsal promesonotum (sometimes), petiolar dorsum, postpetiole and gaster smooth and shiny. Head shape oval to weakly subrectangular, posterior margin widely transverse, sometimes very weakly concave medially, sides convex. Frons partly smooth with relatively long rugula next to short frontal carina. Scapes surpassing posterior head margin by approximately one eye length (SI 105–110), pilosity decumbent to subdecumbent with few suberect hairs along outer edge. Promesonotal outline in profile slightly elongate and flatly convex, with a small process on posterior declivity just anterior of propodeum. Metanotal groove widely impressed, with long, weakly to superficially developed crossribs, interspaces between dorsal cross-ribs smooth and shiny. Propodeum about as high as long, spines relatively long-spinose, almost as long as distance between their bases. Promesonotum dorsally with several short, weakly to faintly developed, transverse rugulae. Metatibia pilosity similar to scape pilosity. Postpetiole on average as long as high (LPpI 100–115), in dorsal view distinctly wider than long (DPpI 121–143). Standing hairs on mesosoma and metasoma not abundant, moderately long, acute, slender, erect to suberect, the two hairs at pronotal humeri very long. Shorter pilosity very fine, subdecumbent to decumbent. Color dark orange to brown, mandibles, antennae, and legs lighter.

**Queens:** Measurements (n = 3): HW 1.11–1.21 (1.17), HL 1.05–1.15 (1.10), SL 0.66–0.73 (0.71), MDL 0.63–0.67 (0.65), EL 0.33–0.35 (0.34), WL 1.64–1.82 (1.75), PNW 1.10–1.18 (1.15), PTL 0.55–0.63 (0.58), PPL 0.34–0.35 (0.35), PTH 0.42–0.46 (0.44), PPH 0.41–0.44 (0.42), PTW 0.37–0.43 (0.40), PPW 0.72–0.74 (0.73), PSL 0.29–0.32 (0.30), MFL 0.97–1.06 (1.02), MTL 0.70–0.78 (0.74), CI 104–109 (106), SI 59–61 (60), MDI 54–57 (55), EI 29–30 (29), FI 85–88 (87), PSLI 24–27 (26), LPpI 80–85 (82), DPpI 206–212 (210), PpWI 172–195 (184), PpHI 92–100 (96). Head slightly wider than long (CI 104–109), anterior margin of clypeus with distinct concave median notch. Mandibles laterally near their bases weakly rugulose. Face mostly smooth between rugae, anterolaterally superficially punctate, rugae often ending before reaching posterior head margin, if reaching the posterior margin then without reticulation. Ventral side of head smooth with some superficial rugulae. Scapes moderately short (SI 59–61), pilosity decumbent to subdecumbent with longer erect hairs along outer edge. Eyes moderately large (EI 29–31). Hypostomal margin with relatively small and reduced median tooth and submedian teeth. Oblique carinae on scutum in anterodorsal view not reaching anterior margin and sometimes reduced or inconspicuous. Lateropronotum, posterior half of anepisternum and propodeum finely rugulose, remainder of mesosoma smooth. Petiole ventrally convex, superficially punctate and sometimes with transverse rugae, anterodorsally smooth. Postpetiole in profile with convex, superficially punctate ventral process, in dorsal view smooth, wide (PpWI 172–195), with lateral corners extended into bluntly angulate processes. Gaster smooth. Standing hairs yellow, abundant, moderately long, erect, with shorter suberect pilosity in between. Color reddish brown, with various dark patches on mesosoma, gaster darker, legs and antennae lighter colored.

Biogeography & Ecology: *Pheidole knowlesi* occurs on most of the major Fijian islands, but was not found on Beqa, Lakeba and Moala. It was collected in primary rainforest, bryophyte forest, secondary forest, along the forest edge, and in disturbed habitat, from sifted litter, malaise traps and general hand collection in elevations between 50 and 1300m. Workers of this species were foraging on the ground, on rocks, on bryophyte plants, and the nests were found under rocks, in logs, twigs, in litter deposits on tree trunks, and in fallen ant-plants.

Comments: The workers of this species are similar to those of *P*. *wilsoni* and *P*. *ululevu*. The major worker posterolateral head corners are normally smooth with very little sculpture, the dorsal head outline in profile varies from convex to weakly impressed between the frontal area and the posterolateral lobes and between the longitudinal rugae, and at antennal scrobes punctures are either absent or only weakly developed. The extent of frontal carinae and longitudinal rugae reaching towards the posterior head margin seems to be in direct correlation to the size and proportion of the head. Major workers with a higher CI and square head often have longer rugae relative to the size of the head than specimens with a longer, more rectangular head shape. Oftentimes major workers of the former type were collected together with those of the latter type in the same sample and from the same colony. Major workers of *P*. *knowlesi* are best separated from the other species by absence of punctures on posterolateral lobes and branching or reticulate rugae between eyes and antennal insertions. The minor workers are best recognized by a low promesonotal profile, the presence of weak or faint longitudinal rugulae on frons and transverse rugulae on dorsal promesonotum and the absence of well-defined punctures on frons and dorsal promesonotum.

#### *Pheidole ululevu* Fischer, Sarnat and Economo sp. n

**Fig 9:**
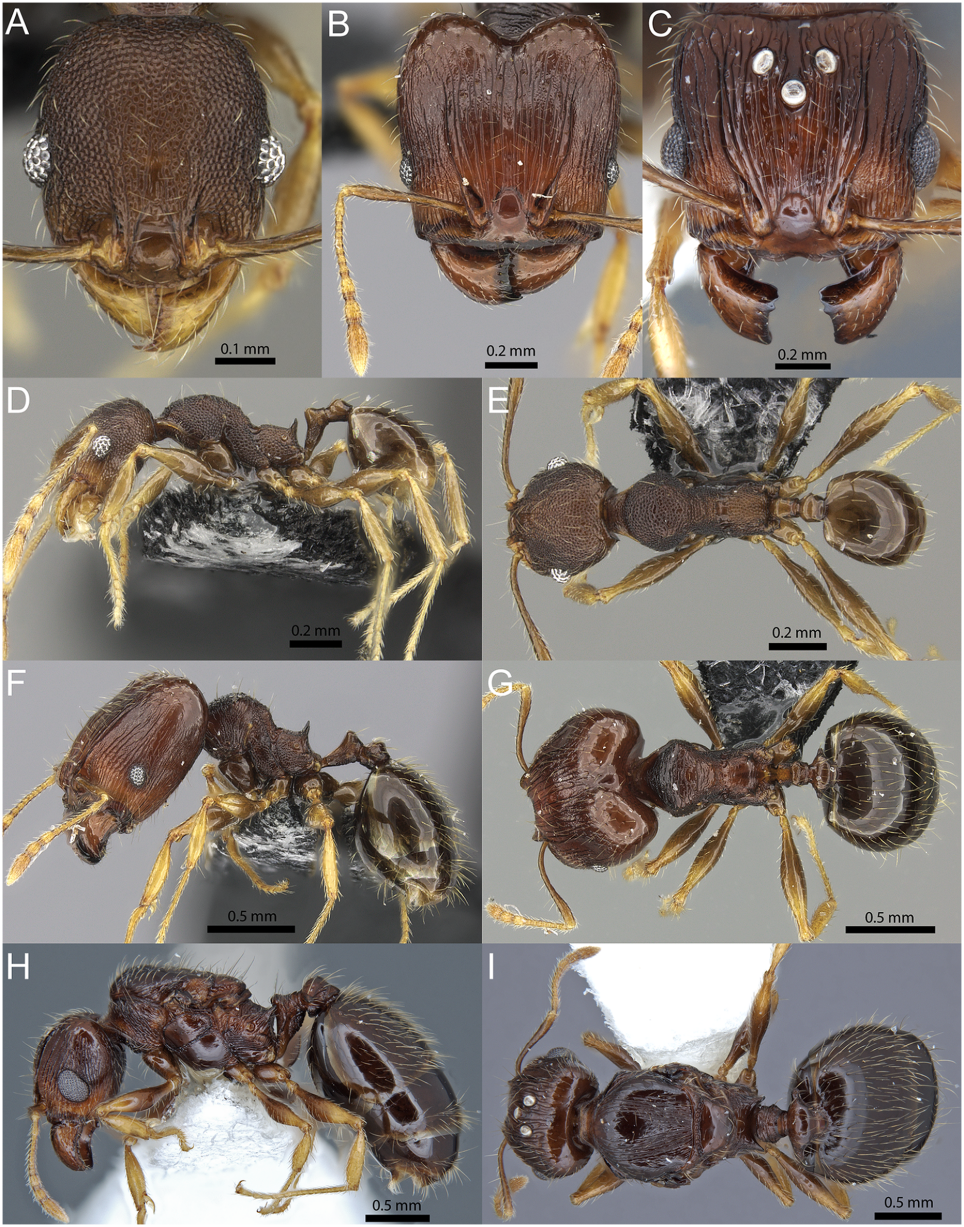
*Pheidole ululevu* sp. n. Minor worker (paratype, CASENT0184114): full-face view (A), profile (D), dorsal view (E). Major worker (holotype, CASENT0184180): full-face view (B), profile (F), dorsal view (G). Queen (CASENT0185564): full-face view (C), profile (H), dorsal view (I).

(Figs 3K–3M and 9, S10–S12 Figs, S10–S12 Vids) [urn:lsid:zoobank.org:act:37467909-2EFD-4647-BE75-70715FF5B158]

This species was previously treated as morphospecies *P*. FJ09 in the following publications: Sarnat & Economo 2012 [2], Sarnat & Moreau 2011 [23], Economo & Sarnat 2012 [24], Economo et al. 2015a [25] & 2015b [26].

**Holotype:** Major worker (BPBM, specimen code CASENT0184180), FIJI, Koro, Mt. Kuitarua, 3.7 km NW Nasau Village, -17.2908, 179.402, 470m, primary forest, nesting in log, 20.vi.2005 (*E*.*M*. *Sarnat*).

**Paratypes:** 4 major workers (MCZC, CASENT0184075; USNM, CASENT0184207; CASENT0184015; CASENT0184187), 3 minor workers (BPBM, CASENT0184114; MCZC, CASENT0184129; USNM, CASENT0184225), same data as holotype.

Other material examined: **FIJI: Beqa:** Malovo 182; Mt. Korovou 326. **Gau:** Navukailagi 300, 408, 415, 432, 475, 480, 490, 496, 505, 535, 564, 597. **Kadavu:** Daviqele 300; Moanakaka 60, 128; Namalata 50, 120; Namara 300; Vunisea 200. **Koro:** Mt. Kuitarua 380; Mt. Nabukala 520; 440 b; Nasau 465 a, 470 (3.7 km); Nasoqoloa 300. **Lakeba:** Tubou 100 b, 100 c. **Moala:** Mt. Korolevu 300; 375. **Ovalau:** Draiba 300; Levuka 400; 450. **Taveuni:** Devo Peak 1187 b; Koronibuabua 235; Lavena 217, 229, 234, 235, 300; Mt. Devo 892; Qacavulo Point 300; Soqulu Estate 140; Tavoro Falls 160. **Vanua Levu:** Drawa 270; Kasavu 300; Kilaka 61; Mt. Delaikoro 699; Mt. Vatudiri 570, 641; Mt. Wainibeqa 152 c; Nakanakana 300; Nakasa 300; Rokosalase 143, 180; Vusasivo Village 400 b; Vuya 300; Yasawa 300. **Viti Levu:** Acaba 700; Colo-i-Suva 186 d, 200, 340 b; Colo-i-Suva Forest Park 220; Galoa 300; Lami 200, 300, 304; Mt. Batilamu 1125 b; Mt. Evans 700; Mt. Korobaba 300, 301; Nabukavesi 300; Nabukelevu 300; Nabutini 300; Naikorokoro 300; Nakavu 200, 300; Nakobalevu 340; Nausori; Navai 1020; Savione 650, 750 b; Vunisea 300. **COOK ISLANDS:** Rarotonga, summit of Te Manga, -21.236, -159.764, 645m, montane rainforest/beating vegetation, 02.x.2010 (*N*. *Porch*).

Diagnosis within the *knowlesi* group: **Major worker:** head usually superficially punctate, on frons and sides with thin longitudinal rugulae that end before reaching the posterior head margin, posterolateral lobes smooth and shiny; anterior clypeal margin with shallow median notch; in profile, head with shallow impression between frons and posterolateral lobes. **Minor worker:** promesonotal profile relatively low, convex, with promesonotal declivity sloping gently towards propodeum; sculpture on dorsal and ventral head and mesosoma uniformly punctate. **Queen:** moderately large (WL 1.32–1.44), legs longer (FI 81–85); clypeus anteriorly with median notch, head sculpture between rugae weakly punctate, rugae fading to weaker rugulae near posterior head margin; carinae on scutum usually reaching its anterior margin; gaster smooth without sculpture anteriorly.

**Major workers:** Measurements (n = 7): HW 0.86–1.18 (1.03), HL 0.91–1.28 (1.11), SL 0.58–0.64 (0.62), MDL 0.47–0.64 (0.56), EL 0.16–0.19 (0.17), WL 0.79–0.94 (0.89), PNW 0.45–0.59 (0.52), PTL 0.30–0.41 (0.34), PPL 0.15–0.20 (0.17), PTH 0.17–0.24 (0.20), PPH 0.15–0.22 (0.19), PTW 0.14–0.20 (0.17), PPW 0.20–0.41 (0.30), PSL 0.14–0.18 (0.16), MFL 0.68–0.81 (0.77), MTL 0.48–0.58 (0.54), CI 92–95 (94), SI 53–67 (60), MDI 52–55 (54), EI 16–19 (17), FI 68–79 (74), PpI 43–69 (57), LPpI 83–100 (90), DPpI 130–210 (171), PpWI 144–210 (178), PpHI 88–100 (95). Ground sculpture on dorsum of head, mesonotum and propodeum weakly to superficially punctate. Remainder of mesosoma and lateral petiole weakly punctate, promesonotum often with irregular transverse rugulae present. Posterolateral lobes and posterior margin of head, petiole and postpetiolar dorsum, and gaster smooth and shiny. Head rectangular with very weakly convex sides, longer than wide (CI 92–95) and posteriorly slightly wider than anteriorly. Mandibles and clypeus medially smooth and shiny, anterior margin of clypeus with shallow median notch. In profile head usually weakly impressed between frons and posterolateral lobes, but in some specimens impression absent or inconspicuous. Frons and sides of head with irregular, interrupted longitudinal rugae, frontal carinae relatively long, often reaching almost towards posterior head margin, posterolateral lobes sometimes with short scattered rugulae. Scapes relatively long (SI 53–67), reaching ca. 2/3 towards posterior head margin, with short decumbent to subdecumbent pilosity and several longer suberect to erect hairs along outer edge. Hypostomal margin with small to medium sized median tooth and submedian teeth. Promesonotum in profile convex, posterior declivity with blunt angle. Metanotal groove weakly impressed, propodeum usually as high as long, sometimes higher. Propodeal spines relatively long, bluntly spinose, and slightly shorter than distance between their bases, oblique to almost vertical. Promesonotal dorsum with several transverse rugulae. Sculpture on anterolateral pronotum, katepisternum, propodeal dorsum and posterior declivity sometimes partly reduced or smooth and shiny. Metatibia pilosity mostly decumbent with few longer suberect hairs along outer edge. Postpetiole with moderately long lateral processes, on average 1.8 times wider than petiole (PpWI 156–194) and 1.7 times wider than long (DPpI 156–184). Standing hairs abundant, suberect, from short and flexuous to moderately long and stiff, shorter pilosity from decumbent to erect.

**Minor workers:** Measurements (n = 7): HW 0.44–0.54 (0.48), HL 0.51–0.61 (0.55), SL 0.51–0.63 (0.58), MDL 0.31–0.38 (0.34), EL 0.11–0.13 (0.12), WL 0.63–0.75 (0.68), PNW 0.30–0.37 (0.33), PTL 0.23–0.28 (0.25), PPL 0.09–0.11 (0.10), PTH 0.12–0.14 (0.13), PPH 0.10–0.12 (0.11), PTW 0.08–0.11 (0.09), PPW 0.10–0.13 (0.12), PSL 0.09–0.13 (0.10), MFL 0.53–0.65 (0.58), MTL 0.38–0.47 (0.41), CI 83–89 (87), SI 109–130 (121), MDI 69–76 (70), EI 20–29 (25), FI 116–131 (121), PpI 32–41 (36), LPpI 86–105 (95), DPpI 100–136 (120), PpWI 123–145 (131), PpHI 71–88 (82). Head and mesosoma uniformly punctate, often overlain with irregular reticulum. Petiole lateroventrally weakly punctate. Mandibles, clypeus, petiolar dorsum, postpetiole and gaster smooth and shiny. Head shape oval to slightly elongate subrectangular, sides weakly convex, posterior margin relatively narrow and concave, with rounded corners. Scapes surpassing posterior head margin by length of preapical antennal segment, scape pilosity decumbent to subdecumbent with few suberect hairs along outer edge. Promesonotal outline in profile relatively flatly convex, metanotal groove widely impressed, laterally with some crossribs. Propodeum as high as long, spines relatively long-spinose and acute, sometimes slightly curved forward, almost as long as distance between their bases. Metatibia pilosity mostly decumbent to subdecumbent. Postpetiole lower than petiole (PpHI 71–88), about as long as high (LPpI 86–105), in dorsal view usually wider than long (DPpI 100–136). Standing hairs on head and mesosoma mostly suberect, moderately long and abundant, not as slender and flexuous as in other species of this group. Pilosity shorter, relatively coarse with blunt apices, mostly subdecumbent to suberect. Head and mesosoma brown to dark brown, mandibles, antennae, legs, waist segments and gaster lighter colored.

**Queens:** Measurements (n = 3): HW 0.94–1.03 (0.97), HL 0.90–0.97 (0.93), SL 0.62–0.65 (0.64), MDL 0.52–0.55 (0.54), EL 0.28–0.30 (0.29), WL 1.32–1.44 (1.38), PNW 0.86–1.00 (0.93), PTL 0.47–0.52 (0.50), PPL 0.25–0.29 (0.27), PTH 0.32–0.33 (0.33), PPH 0.32–0.34 (0.33), PTW 0.25–0.31 (0.28), PPW 0.54–0.60 (0.57), PSL 0.21–0.25 (0.23), MFL 0.78–0.83 (0.81), MTL 0.56–0.62 (0.59), CI 104–106 (105), SI 62–68 (66), MDI 52–59 (55), EI 29–31 (30), FI 81–85 (83), PSLI 22–24 (24), LPpI 76–85 (81), DPpI 207–232 (216), PpWI 193–232 (206), PpHI 100–103 (101). Head slightly wider than long (CI 104–106), anterior margin of clypeus with distinct median notch. Face weakly to superficially punctate anteriorly, almost smooth between rugae and near posterior head margin. Rugulae posteriorly sometimes weakly reticulate, ventral side of head rugulose. Scapes moderately short (SI 62–68), pilosity mostly decumbent with longer suberect hairs along outer edge. Hypostomal margin with well-developed median tooth and submedian teeth of about same size. Oblique carinae on scutum usually reaching anterior margin in anterodorsal view, remainder of scutum smooth to superficially punctate. Scutellum, most of anepisternum and katepisternum smooth, lateropronotum weakly rugulose to rugopunctate. Propodeum laterally with abundant, well-defined oblique, curved rugulae. Petiole laterally and ventrally weakly punctate, often with weak lateral carina that runs around posterior face of petiolar node. Postpetiole in profile with very convex and punctate ventral process, in dorsal view smooth, wide, with lateral corners extended into blunt processes. Gaster smooth. Standing hairs of varying lengths, longer on scutellum, abundant, mostly suberect, yellow. Color brown or dark brown, anterior of head, mandibles, antennae and legs lighter colored, flagella, tibia, and tarsi frequently bright yellow.

Biogeography & Ecology: *Pheidole ululevu* sp. n. is the most widespread species in the *knowlesi* group, with a distribution across all sampled islands. A single disjunct population was recently discovered more than two thousand kilometers east on the summit of Te Manga peak on Rarotonga island. Te Manga is the highest mountain on the entire island, thus making this distinct population extremely vulnerable to warming trends caused by global climate change. On Fiji the species was collected in leaf litter, inside dead branches and logs, in the vegetation, foraging on logs, on trees and under bark of fallen trees, nesting under stones and in epiphyte soil, and in malaise traps. *Pheidole ululevu* was found in disturbed, mahogany, logged, secondary, bryophyte, coastal forest, primary rainforest, lowland rainforest, from elevations between 50 and 1187m.

Comments: This species is closely related to *P*. *wilsoni* and *P*. *knowlesi* and its major workers can readily be distinguished from these two by the presence of mostly longitudinal, rarely branching rugulae posterior of the antennal scrobe and mostly punctate sculpture on the metapleuron. While the majors of *P*. *wilsoni* have several to many branching and reticulate rugulae posterior to the antennal scrobe and mesosoma sculpture mostly smooth to weakly punctate, the head and mesosoma of *P*. *knowlesi* majors are much more shiny and have distinctly less punctate and rugulose sculpture, between the rugae on frons, posterolateral lobes and on the mesosoma. Minor workers of *P*. *ululevu* can be easily separated from the other species by their uniformly punctate sculpture on head (dorsal & ventral) and mesosoma. The workers collected from Colo-i-Suva on Viti Levu differ from other populations in possessing a glassy smooth ground sculpture. Morphologically, *P*. *ululevu* is very similar to *P*. *kava* and can be distinguished from the latter with the characters given in the diagnosis and also in the discussion under *P*. *kava*.

Etymology: This species’ epithet is derived from the Fijian language and consists of the words *ulu* for ‘head’ and *levu* for ‘large’. The name is a noun in apposition and thus invariant.

#### *Pheidole vatu* Mann

**Fig 10:**
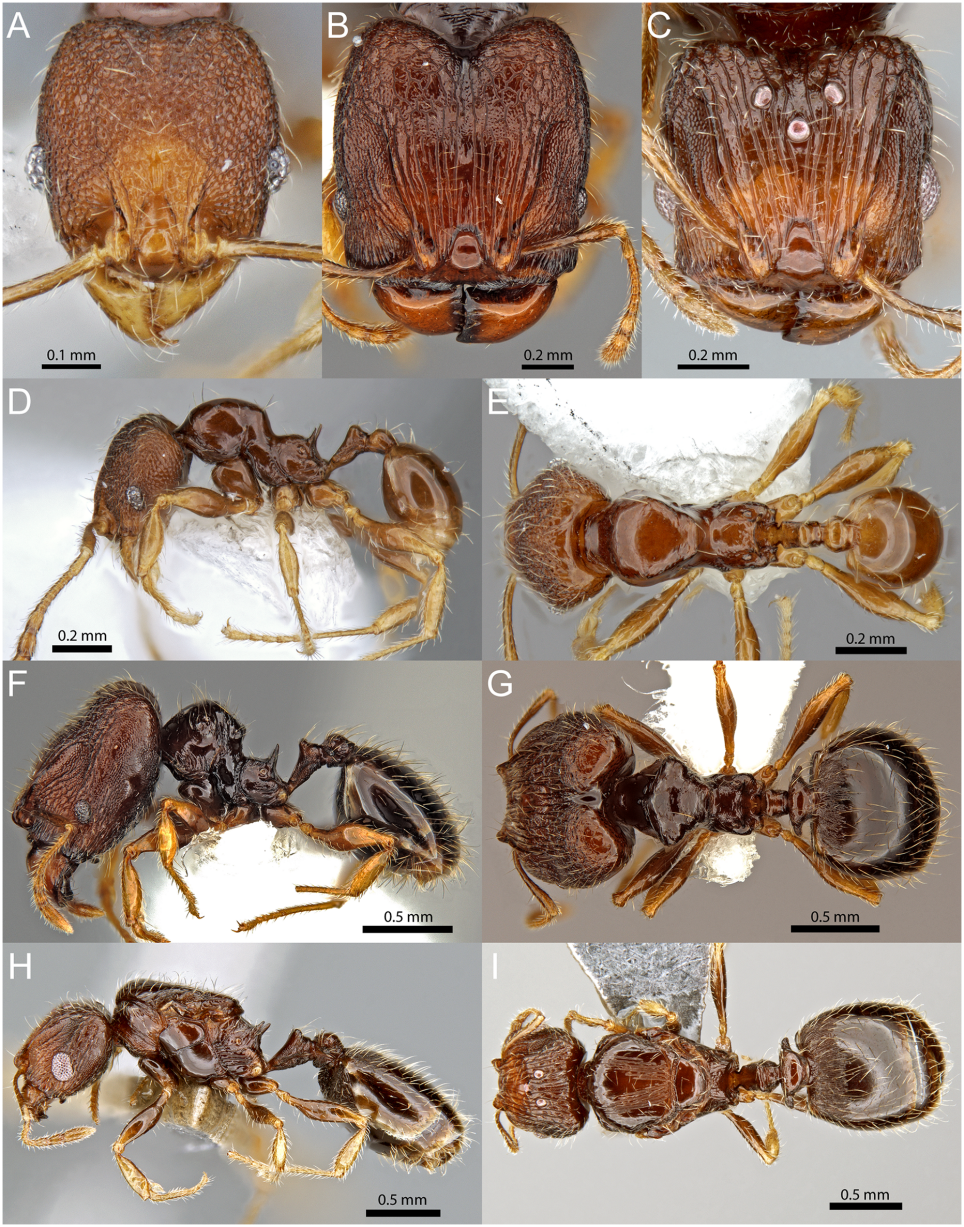
*Pheidole vatu* Mann. Minor worker (CASENT0183692): full-face view (A), profile (D), dorsal view (E). Major worker (CASENT0184486): full-face view (B), profile (F), dorsal view (G). Queen (CASENT0185452): full-face view (C), profile (H), dorsal view (I).

(Figs 3N–3P and 10, S13–S15 Figs, S13–S15 Vids)

*Pheidole vatu* Mann, 1921 [17]: 431, fig. 13b; major worker, minor worker described. Type locality: FIJI, Viti Levu, Nadarivatu (W. M. Mann). Syntypes: 6 minor workers, 6 major workers (MCZC type no. 8695, examined).

Material examined: **Gau:** Navukailagi 300, 356, 415, 432. **Kadavu:** Mt. Washington 700. **Koro:** Nasau 420 b, 465 a. **Moala:** Mt. Korolevu 300, 375. **Ovalau:** Draiba 300. **Taveuni:** Lavena 300. **Vanua Levu:** Kasavu 300; Kilaka 146; Nakanakana 300; Yasawa 300. **Viti Levu:** Colo-i-Suva 186 d; Galoa 300; Korobaba 300; Lami 200, 300; Monasavu Dam 800; Mt. Rama 300; Naboutini 300; Nabukavesi 300; Nabukelevu 300; Nadarivatu 750; Naikorokoro 300; Nakavu 300; Nakobalevu 340; Nasoqo 800 d; Navai 700, 863, 930, 1000; Waimoque 850; Waivudawa 300; Vatubalavu 300.

Diagnosis within the *knowlesi* group: **Major worker:** head weakly to superficially punctate between rugae, scrobes broad and completely punctate, posterolateral lobes rugoreticulate; clypeus medially notched; in profile head dorsum posteriorly of frons impressed; humeri projecting obtusely laterally; gaster anteriorly widely punctate. **Minor worker:** head completely punctate, but mesosoma largely smooth, at least with large smooth areas present on lateral pronotum, posterior declivity of promesonotum, and on mesopleuron; promesonotal dome strongly convex in profile, with relatively steep and high posterior declivity. **Queen:** small (WL 1.10–1.16), legs shorter (FI 77–79); anterior clypeal margin with median notch present, longitudinal rugae reaching posterior hear margin, area between rugae superficially punctate on frons, scrobe and sides of head punctate, ventrally of scrobes and posterior head margin weakly reticulate, gaster anterodorsally rugulose and anteroventrally strongly reticulate-punctate.

**Major workers:** Measurements (n = 7): HW 0.73–1.02 (0.90), HL 0.83–1.07 (0.97), SL 0.39–0.50 (0.47), MDL 0.42–0.54 (0.49), EL 0.12–0.14 (0.13), WL 0.69–0.82 (0.77), PNW 0.43–0.53 (0.48), PTL 0.27–0.35 (0.31), PPL 0.13–0.18 (0.16), PTH 0.17–0.21 (0.19), PPH 0.16–0.21 (0.18), PTW 0.14–0.17 (0.16), PPW 0.28–0.42 (0.34), PSL 0.12–0.16 (0.14), MFL 0.46–0.60 (0.56), MTL 0.34–0.45 (0.41), CI 88–96 (93), SI 48–59 (53), MDI 51–58 (55), EI 13–16 (14), FI 59–67 (62), PpI 60–79 (69), LPpI 76–100 (88), DPpI 175–242 (208), PpWI 176–247 (212), PpHI 94–100 (98). Ground sculpture on head, sides of petiole and postpetiole, anterior dorsum and venter of gaster uniformly weakly punctate. Mesosoma, dorsal petiole and postpetiole, and posterior gaster smooth and shiny. Head subrectangular, head narrower at anterior margin and at posterior corners, widest point behind frons and posterior 1/3 of sides oblique. Mandibles smooth, clypeus smooth and with very fine microsculpture, anterior margin with median notch. Antennal pit moderately excavated. Frons weakly convex in profile view, head impressed between frons and posterolateral lobes. Frontal carinae and longitudinal rugae moderately short, ending about half way between posterior eye level and posterior head margin, the posterior 1/3 of head covered with rugoreticulum. Antennal scrobe weakly defined, punctate, and weakly depressed at posterior end. Posteriorly of frons and laterally of scrobes rugae weakly reticulate. Median hypostomal tooth reduced to absent, submedian teeth small. Promesonotum in profile with a simple, convex dome, declivity angulate and posteriorly vertical. Metanotal groove not impressed. Propodeum short, significantly higher than long in profile, with relatively long, almost vertical spines. In dorsal view pronotal humeri weakly projecting with rounded corners. Mesosoma shining with weakly punctate patches and irregular transverse rugulae on pronotum. Laterally with moderately to relatively large smooth areas on pronotum, katepisternum and sometimes propodeum. Metatibia pilosity fine, decumbent with one or two subdecumbent hairs along outer edge. Standing hairs suberect, moderately long, flexuous and abundant, on gaster longer and with a relatively thicker base, shorter pilosity mostly subdecumbent. Colour dark brown, head and mandibles lighter reddish brown, funniculi and legs brown.

**Minor workers:** Measurements (n = 7): HW 0.41–0.47 (0.44), HL 0.43–0.51 (0.47), SL 0.35–0.45 (0.41), MDL 0.25–0.32 (0.27), EL 0.09–0.11 (0.10), WL 0.50–0.59 (0.54), PNW 0.28–0.31 (0.29), PTL 0.19–0.22 (0.21), PPL 0.08–0.10 (0.09), PTH 0.10–0.11 (0.10), PPH 0.08–0.10 (0.09), PTW 0.08–0.09 (0.08), PPW 0.11–0.14 (0.12), PSL 0.08–0.11 (0.09), MFL 0.35–0.44 (0.40), MTL 0.25–0.32 (0.28), CI 90–96 (93), SI 85–96 (93), MDI 58–68 (62), EI 21–23 (22), FI 85–96 (92), PpI 36–48 (41), LPpI 100–114 (105), DPpI 117–156 (133), PpWI 137–156 (146), PpHI 80–90 (85). Head dorsally and ventrally densely punctate. Petiole laterally moderately punctate. Majority of mesosoma varying from weakly punctate to smooth and shiny, but always with large smooth areas on lateral promesonotum, mesopleuron and promesonotal posterior declivity. Dorsum of petiole, postpetiole and gaster smooth and shiny. Head in full-face view subrectangular, slightly longer than wide (CI 90–96), with medially concave posterior margin and convex sides. Eyes small, with 5 ommatidia in the longest row. Scapes barely surpassing posterior head margin (by less than one eye length), scape pilosity decumbent with few, very short suberect hairs along outer edge. Promesonotal outline in profile compact, with weakly convex dorsum and right angle towards steep posterior declivity. Metanotal groove shallowly impressed. Propodeum small and compact, slightly higher than long, spines acute spinose, weakly curved anteriorly, distinctly shorter than distance between their bases. Metatibia pilosity decumbent. Postpetiole lower than petiole (PpHI 80–90), usually slightly longer than high (LPpI 100–114), in dorsal view on average 1.3 times wider than long (DPpI 117–156). Standing hairs sparse, suberect, moderately long, with shorter subdecumbent pilosity in between. Head and body dark brown, mandibles, antennae, and legs lighter colored.

**Queens:** Measurements (n = 2): HW 0.76–0.78 (0.77), HL 0.72–0.75 (0.74), SL 0.48–0.50 (0.49), MDL 0.38–0.43 (0.41), EL 0.21–0.22 (0.22), WL 1.10–1.16 (1.13), PNW 0.71, PTL 0.41, PPL 0.21–0.22 (0.22), PTH 0.25, PPH 0.26–0.28 (0.27), PTW 0.25, PPW 0.50–0.52 (0.51), PSL 0.19–0.20 (0.20), MFL 0.60, MTL 0.45, CI 101–108 (105), SI 63–64 (64), MDI 50–55 (53), EI 27–29 (28), FI 77–79 (78), PSLI 25–26 (25), LPpI 79–81 (80), DPpI 236, PpWI 202–208 (205), PpHI 104–112 (108). Head slightly wider than long (CI 101–108), anterior margin of clypeus with shallow median notch. Scapes moderately short (SI 63–64), pilosity very fine and decumbent to subdecumbent, with one or two longer suberect hairs along outer edge. Eyes moderately small (EI 27–29). Face with longitudinal rugae reaching posterior head margin, ground sculpture on frons superficially punctate, on scrobes and sides of head weakly punctate, areas ventrally and posterior of scrobes and posterior head margin weakly reticulate. Ventral side of head weakly rugopunctate. Hypostomal margin with small median and submedian teeth. Oblique carinae on scutum reaching or almost reaching anterior margin in anterodorsal view. Lateropronotum mostly smooth with few oblique rugulae to weakly rugopunctate and anepisternum dorsolaterally sometimes weakly rugopunctate. Propodeum laterally with several weak rugulae and otherwise smooth. Posterodorsal face of propodeum weakly to superficially rugopunctate, remainder of mesosoma smooth. Petiole ventrally with weak convexity and with few irregular rugulae present on its surface. Anterodorsal face of petiole mostly smooth to mostly punctate and posterior face transversally rugulose. Postpetiole in profile with convex, punctate ventral process. In dorsal view rugulose to mostly smooth, about 2x wider than petiole (PpWI 202–208), with lateral corners extended into blunt to weakly pointed processes. Gaster mostly smooth, anterodorsally widely rugulose, ventrally with first half of sternite quite strongly reticulate-punctate. Standing hairs moderately abundant, relatively short, suberect, and light yellow. Color brown or dark brown, anterior of antennal funiculus, tibiae and tarsi lighter colored to yellow.

Biogeography & Ecology: Except on Viti Levu, where it was found in several localities, *Pheidole vatu* was collected relatively rarely. Yet it occurs on most of the major Fijian islands and only seems to be absent from Beqa and Lakeba. It was collected from leaf litter, under stones, under fallen branches, and from moss on logs, in primary and secondary rainforest, disturbed forest and along the forest edge, in elevations between 146 and 1000m.

Comments: Minor workers of *Pheidole vatu* show considerable variation in extent of punctate sculpture on the dorsal promesonotum, which can vary from uniformly weakly punctate to completely smooth and shiny. This species is the easiest to identify among the *knowlesi* group on Fiji. Morphologically, workers and queens of *P*. *vatu* are most similar to those of *P*. *kava*, both species are slightly smaller than *P*. *knowlesi*, *P*. *vatu* and *P*. *wilsoni*. However, workers of *Pheidole vatu* can be recognized by the presence of large smooth areas on lateral and dorsal mesonotum, many specimens are almost completely smooth and shiny with only small remnants of superficial sculpture. Major and minor workers also have a highly domed promesonotum with a steep posterior declivity.

#### *Pheidole wilsoni* Mann

**Fig 11:**
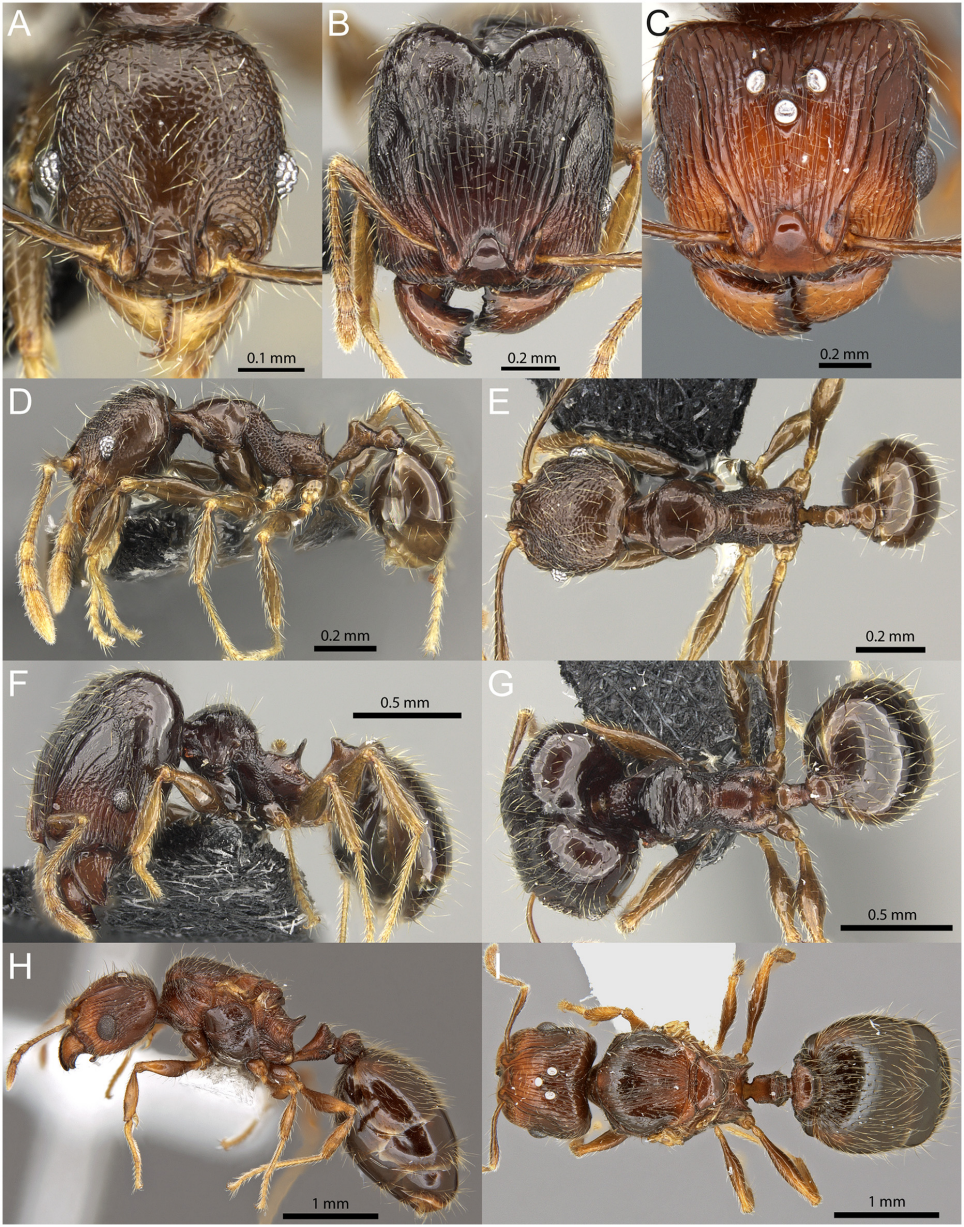
*Pheidole wilsoni* Mann. Minor worker (CASENT0185076): full-face view (A), profile (D), dorsal view (E). Major worker (CASENT0185117): full-face view (B), profile (F), dorsal view (G). Queen (CASENT0184003): full-face view (C), profile (H), dorsal view (I).

(Figs 3Q–3S and 11, S16–S18 Figs, S16–S18 Vids)

*Pheidole wilsoni* Mann, 1921 [17]: 433; major worker, minor worker described. Type locality: FIJI, Kadavu, Vanua Ava (W. M. Mann). Syntypes: 3 minor workers, 3 major workers (USNM, not examined); (MCZC 8696, not examined).

Material examined: **Gau**: Navukailagi 356, 408, 415, 432, 475, 480, 496, 505, 575, 625. **Kadavu:** Daviqele 300; Mt. Washington 700, 760; Namara 300; Vunisea 200. **Lakeba:** Tubou 100 c. **Moala:** Mt. Korolevu 300. **Ovalau:** Draiba 300; Levuka 450. **Taveuni:** Lavena 300; Tavoro Falls 160. **Vanua Levu:** Drawa 270; Kasavu 300; Labasa; Lagi 300; Mt. Delaikoro 391, 699, 910; Mt. Vatudiri 570, 641; Nakanakana 300; Nakasa 300; Vusasivo Village 400 a, 400 b; Vuya 300; Yasawa 300. **Viti Levu:** Colo-i-Suva 186 d, 200, 340 b; Galoa 300; Korobaba 300; Lami 171, 200, 300, 304; Monasavu 850; Mt. Batilamu 840 c; Mt. Evans 700, 800; Mt. Naqaranabuluti 912, 1050; Mt. Rama 300; Mt. Tomanivi 950; Naboutini 300; Nabukavesi 300; Nabukelevu 300; Nadakuni 300, 300 b, 340; Nadala 300; Naikorokoro 300; Naitasiri 300; Nakavu 200; Nakobalevu; Naqaranabuluti 860, 1000; Navai 930; Savuione 800; Vatubalavu 300; Vaturu Dam 575 b; Vunisea 300; Nausori.

Diagnosis within the *knowlesi* group: **Major worker:** moderately large (WL 0.87–0.97); head in profile weakly impressed between frontal area and posterolateral lobes; frontal carina sharply defined, scrobe posteriorly bordered by transverse to arcuate rugulae; rugulae on posterior third of head usually branching. **Minor worker:** head dorsum punctate, punctures medially partly effaced, pronotum and head venter smooth to superficially punctate; promesonotal outline in profile flatly convex, posterior declivity declining evenly. **Queen:** moderately large (WL 1.40–1.48), with longer legs (FI 82–86); clypeus with median notch; median hypostomal tooth distinctly smaller than submedian teeth; rugulae on mesosoma dorsum usually reaching anterior margin of scutum; gaster anterodorsally, near postpetiole, rugopunctate.

**Major workers:** Measurements (n = 7): HW 1.02–1.19 (1.15), HL 1.10–1.28 (1.21), SL 0.61–0.65 (0.63), MDL 0.55–0.65 (0.63), EL 0.14–0.18 (0.15), WL 0.87–0.97 (0.91), PNW 0.53–0.55 (0.54), PTL 0.35–0.43 (0.39), PPL 0.17–0.20 (0.19), PTH 0.21–0.24 (0.23), PPH 0.19–0.22 (0.20), PTW 0.15–0.18 (0.17), PPW 0.26–0.37 (0.33), PSL 0.15–0.20 (0.18), MFL 0.75–0.82 (0.78), MTL 0.51–0.58 (0.54), CI 93–98 (94), SI 51–62 (55), MDI 52–56 (54), EI 12–17 (13), FI 63–80 (68), PpI 48–69 (62), LPpI 89–95 (92), DPpI 153–195 (178), PpWI 178–211 (196), PpHI 85–93 (89). Head and mesosoma with weakly to superficially punctate ground sculpture. Posterolateral lobes with scarce punctures and few short, superficial rugulae present. Dorsum of promesonotum with transverse rugulae. Petiole and postpetiole laterally and ventrally superficially punctate, dorsally smooth and shiny. Gaster smooth and shiny. Head subrectangular, sides convex and curved towards posterior head corners, posteriorly about as wide as anteriorly or slightly narrower. Mandibles and clypeus smooth, anterior margin with shallow median notch. Antennal socket situated in excavated pit, frontal lobes translucent. In profile view dorsal outline of head shallowly impressed between frons and posterolateral lobes. Small median tooth on hypostomal margin flanked by comparatively large submedian teeth. Moderately well-developed longitudinal rugae on frons ending before posterior head margin and posterolateral lobes. Frontal carina sharply defined and raised, bordering whole length of scrobe, reaching posterior end of scapes when in repose and reaching about 3/4 towards posterior head margin. Scrobe usually well-defined and wide. Scape length more than 1/2 of head width (SI 55–67), slightly surpassing anterior 2/3 of head, with decumbent pilosity and longer erect hairs along outer edge. Promesonotum in profile convex with steep, angulate posterior declivity. Metanotal groove not or very shallowly impressed. Propodeum in profile slightly higher than long, its dorsal face shorter than posterior declivity. Spines about as long as distance between their bases. In dorsal view pronotal humeri not projecting laterally. Metatibia pilosity mostly decumbent with subdecumbent longer hairs along outer edge. Postpetiole laterally with short processes, ventrally superficially punctate, and dorsally smooth and shiny. Standing hairs abundant, moderately long and flexuous, shorter pilosity decumbent to subdecumbent. Colour dark brown, mandibles, antennae and legs brown to apically light brown.

**Minor workers:** Measurements (n = 7): HW 0.48–0.55 (0.52), HL 0.53–0.62 (0.58), SL 0.55–0.65 (0.61), MDL 0.33–0.40 (0.36), EL 0.11–0.13 (0.12), WL 0.64–0.79 (0.71), PNW 0.32–0.37 (0.35), PTL 0.24–0.29 (0.26), PPL 0.10–0.13 (0.12), PTH 0.13–0.15 (0.14), PPH 0.11–0.13 (0.12), PTW 0.08–0.12 (0.10), PPW 0.13–0.17 (0.15), PSL 0.09–0.13 (0.11), MFL 0.57–0.66 (0.62), MTL 0.40–0.48 (0.45), CI 86–91 (89), SI 113–120 (118), MDI 66–74 (69), EI 22–25 (23), FI 115–122 (120), PpI 39–45 (42), LPpI 86–108 (97), DPpI 113–150 (128), PpWI 131–160 (143), PpHI 79–83 (82). Head, mesonotum, lateral propodeum, and lateral petiole punctate to weakly punctate. Punctures on sides of face stronger and often overlain with weak, irregular rugulae. Head venter, majority of promesonotum, dorsal petiole and postpetiole, and gaster smooth and shiny. Head shape oval, with strongly convex sides and moderately wide, medially weakly concave posterior margin, and corners rounded. Mandibles smooth and clypeus smooth with short carinae anteriorly or laterally. Scapes surpassing posterior head margin by length of preapical antennal segment, pilosity subdecumbent with few suberect hairs along outer edge. Promesonotal outline in profile flatly convex, with evenly oblique posterior declivity. Metanotal groove relatively shallow, crossribs weakly developed to inconspicuous. Propodeum about as high as long to slightly lower. Spines curved forward, relatively long-spinose and acute, shorter than distance between their bases. Promesonotum dorsum often with faint traces of short, transverse rugulae and superficial microsculpture. Metatibia pilosity decumbent on inner edge and subdecumbent along outer edge. Postpetiole lower than petiole (PpHI 79–83), about as long as high (LPpI 86–108), in dorsal view distinctly wider than long (DPpI 113–150). Standing hairs on head and mesosoma mostly suberect, moderately long and abundant, slender, several hairs inclined in opposing directions. Shorter pilosity very fine, mostly subdecumbent. Colour brown to dark brown, mandibles, antennal funiculus, and legs lighter colored.

**Queens:** Measurements (n = 3): HW 1.03–1.08 (1.05), HL 0.95–1.00 (0.97), SL 0.66–0.71 (0.68), MDL 0.55–0.61 (0.58), EL 0.27–0.28 (0.27), WL 1.40–1.48 (1.43), PNW 1.00–1.03 (1.02), PTL 0.45–0.57 (0.51), PPL 0.25–0.28 (0.26), PTH 0.36–0.38 (0.37), PPH 0.34–0.37 (0.35), PTW 0.30–0.33 (0.32), PPW 0.59–0.62 (0.61), PSL 0.26–0.28 (0.27), MFL 0.85–0.89 (0.88), MTL 0.58–0.63 (0.61), CI 108, SI 63–68 (65), MDI 53–56 (55), EI 25–27 (26), FI 82–86 (84), PSLI 24–27 (25), LPpI 71–77 (74), DPpI 221–248 (235), PpWI 179–207 (193), PpHI 94–97 (95). Head slightly wider than long (CI 108), anterior margin of clypeus with median notch. Scapes moderately short (SI 63–68), pilosity decumbent with longer suberect hairs along outer edge. Eyes relatively small (EI 25–27). Frons smooth, scrobes and sides of head weakly punctate, longitudinal rugae reaching posterior head margin, area above scrobes weakly reticulate, ventral side of head rugulose. Hypostomal margin with small median tooth and two distinctly larger submedian teeth. Oblique carinae on scutum usually reaching anterior margin in anterodorsal view. Lateropronotum obliquely rugulose and anepisternum dorsolaterally sometimes weakly rugopunctate. Propodeum laterally with abundant, well-defined curved rugulae, dorsally usually effaced. Remainder of mesosoma smooth. Petiole ventrally with convex bulge, with several transverse raised rugae present on its surface. Anterodorsal face of petiole smooth to punctate and posterior face transversally rugulose. Postpetiole in profile with very convex and punctate ventral process, in dorsal view anteriorly rugopunctate and posteriorly smooth, wide (PpWI 179–207), with lateral corners extended into blunt processes. Gaster mostly smooth, but anteriorly near postpetiole, rugopunctate. Standing hairs relatively abundant, of varying lengths, suberect to subdecumbent, yellow. Colour brown or dark brown, anterior of head, mandibles, antennae and legs lighter coloured to yellow.

Biogeography & Ecology: *Pheidole wilsoni* has a widespread distribution across the Fiji island archipelago and was collected on most of the sampled islands, except Beqa and Koro. It seems to be an ecologically versatile species and was collected from leaf litter, nesting under stones, in sticks, rotten logs, in ant-plants and under moss in trees. *Pheidole wilsoni* can be found in several forest types, from primary rainforest to disturbed, logged, and fragmented forest, and occurs in elevations between 100 and 1050m.

Comments: *Pheidole wilsoni* is phylogenetically and morphologically closely related to *P*. *knowlesi* and *P*. *ululevu* and the characters to distinguish them are listed in the diagnosis and discussed in the other two species. As in the other species in this group, with the possible exception of *P*. *kava*, the major workers of *P*. *wilsoni* show a notable variation in total head size when viewed in proportion to total body size, with the larger specimens often having more robustly developed posterolateral lobes and thus a more elongate head shape.

## Supporting Information

S1 Fig *Pheidole caldwelli* Mann. 3D pdf of volumetric surface model (major worker, CASENT0709599). (When viewing the 3D pdfs with Adobe Acrobat Reader (version 8 or higher), trusting the document by clicking on the image will activate the interactive 3D-mode and allows rotating, moving and zooming into the model.)
(PDF)

S2 Fig *Pheidole caldwelli* Mann. 3D pdf of volumetric surface model (minor worker, CASENT0709600). (PDF)

S3 Fig *Pheidole caldwelli* Mann. 3D pdf of volumetric surface model (queen, CASENT0185623). (PDF)

S4 Fig *Pheidole kava* sp. n. 3D pdf of volumetric surface model (major worker, CASENT0185437). (PDF)

S5 Fig *Pheidole kava* sp. n. 3D pdf of volumetric surface model (minor worker, CASENT0183982). (PDF)

S6 Fig *Pheidole kava* sp. n. 3D pdf of volumetric surface model (queen, CASENT0194645). (PDF)

S7 Fig *Pheidole knowlesi* Mann. 3D pdf of volumetric surface model (major worker, CASENT0183992). (PDF)

S8 Fig *Pheidole knowlesi* Mann. 3D pdf of volumetric surface model (minor worker, CASENT0184016). (PDF)

S9 Fig *Pheidole knowlesi* Mann. 3D pdf of volumetric surface model (queen, CASENT0184086). (PDF)

S10 Fig *Pheidole ululevu* sp. n. 3D pdf of volumetric surface model (major worker, CASENT0183915). (PDF)

S11 Fig *Pheidole ululevu* sp. n. 3D pdf of volumetric surface model (minor worker, CASENT0183250). (PDF)

S12 Fig *Pheidole ululevu* sp. n. 3D pdf of volumetric surface model (queen, CASENT0185564). (PDF)

S13 Fig *Pheidole vatu* Mann. 3D pdf of volumetric surface model (major worker, CASENT0184486). (PDF)

S14 Fig *Pheidole vatu* Mann. 3D pdf of volumetric surface model (minor worker, CASENT0185591). (PDF)

S15 Fig *Pheidole vatu* Mann. 3D pdf of volumetric surface model (queen, CASENT0185452). (PDF)

S16 Fig *Pheidole wilsoni* Mann. 3D pdf of volumetric surface model (major worker, CASENT0184311). (PDF)

S17 Fig *Pheidole wilsoni* Mann. 3D pdf of volumetric surface model (minor worker, CASENT0184378). (PDF)

S18 Fig *Pheidole wilsoni* Mann. 3D pdf of volumetric surface model (queen, CASENT0184003). (PDF)

S1 Vid *Pheidole caldwelli* Mann. Volumetric surface rendering rotational video (major worker, CASENT0709599). (MPG)

S2 Vid *Pheidole caldwelli* Mann. Volumetric surface rendering rotational video (minor worker, CASENT0709600). (MPG)

S3 Vid *Pheidole caldwelli* Mann. Volumetric surface rendering rotational video (queen, CASENT0185623). (MPG)

S4 Vid *Pheidole kava* sp. n. Volumetric surface rendering rotational video (major worker, CASENT0185437). (MPG)

S5 Vid *Pheidole kava* sp. n. Volumetric surface rendering rotational video (minor worker, CASENT0183982). (MPG)

S6 Vid *Pheidole kava* sp. n. Volumetric surface rendering rotational video (queen, CASENT0194645). (MPG)

S7 Vid *Pheidole knowlesi* Mann. Volumetric surface rendering rotational video (major worker, CASENT0183992). (MPG)

S8 Vid *Pheidole knowlesi* Mann. Volumetric surface rendering rotational video (minor worker, CASENT0184016). (MPG)

S9 Vid *Pheidole knowlesi* Mann. Volumetric surface rendering rotational video (queen, CASENT0184086). (MPG)

S10 Vid *Pheidole ululevu* sp. n. Volumetric surface rendering rotational video (major worker, CASENT0183915). (MPG)

S11 Vid *Pheidole ululevu* sp. n. Volumetric surface rendering rotational video (minor worker, CASENT0183250). (MPG)

S12 Vid *Pheidole ululevu* sp. n. Volumetric surface rendering rotational video (queen, CASENT0185564). (MPG)

S13 Vid *Pheidole vatu* Mann. Volumetric surface rendering rotational video (major worker, CASENT0184486). (MPG)

S14 Vid *Pheidole vatu* Mann. Volumetric surface rendering rotational video (minor worker, CASENT0185591). (MPG)

S15 Vid *Pheidole vatu* Mann. Volumetric surface rendering rotational video (queen, CASENT0185452). (MPG)

S16 Vid *Pheidole wilsoni* Mann. Volumetric surface rendering rotational video (major worker, CASENT0184311). (MPG)

S17 Vid *Pheidole wilsoni* Mann. Volumetric surface rendering rotational video (minor worker, CASENT0184378). (MPG)

S18 Vid *Pheidole wilsoni* Mann. Volumetric surface rendering rotational video (queen, CASENT0184003). (MPG)
