# Correction: Revision and Microtomography of the *Pheidole knowlesi* Group, an Endemic Ant Radiation in Fiji (Hymenoptera, Formicidae, Myrmicinae)

**DOI:** 10.1371/journal.pone.0161520

**Published:** 2016-08-16

**Authors:** The PLOS ONE Staff

## Notice of Republication

This article was republished on August 8, 2016, to correct errors in the title and Table 2. The publisher apologizes for the errors. Please download this article again to view the correct version. The originally published, uncorrected article and the republished, corrected article are provided here for reference.

## Supporting Information

S1 File Originally published, uncorrected article. (PDF)

S2 File Republished, corrected article. (PDF)

## References

[1] Fischer G, Sarnat EM, Economo EP (2016) Revision and Microtomography of the *Pheidole knowlesi Group*, an Endemic Ant Radiation in Fiji (Hymenoptera, Formicidae, Myrmicinae). PLoS ONE 11(7): e0158544 doi: 10.1371/journal.pone.0158544 27462877 10.1371/journal.pone.0158544 PMC4963041

