# Supplementary figures and images for: Revision and Microtomography of the Pheidole knowlesi Group, an Endemic Ant Radiation in Fiji (Hymenoptera, Formicidae, Myrmicinae)
Source: PLoS One. 2016 Jul 27;11(7):e0158544. doi: 10.1371/journal.pone.0158544 (PMC4963041; doi:10.1371/journal.pone.0158544)

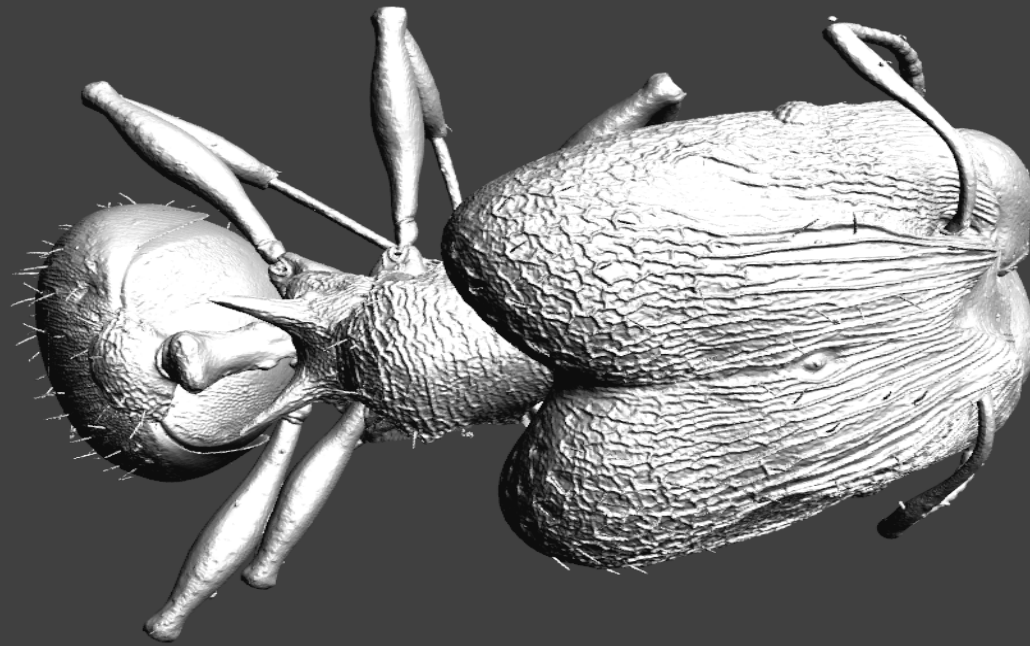

*Pheidole caldwelli*  
major worker CASENT0709599

Supplement: S1 Fig — (When viewing the 3D pdfs with Adobe Acrobat Reader (version 8 or higher), trusting the document by clicking on the image will activate the interactive 3D-mode and allows rotating, moving and zooming into the model.) (PDF) [file pone.0158544.s001.pdf]

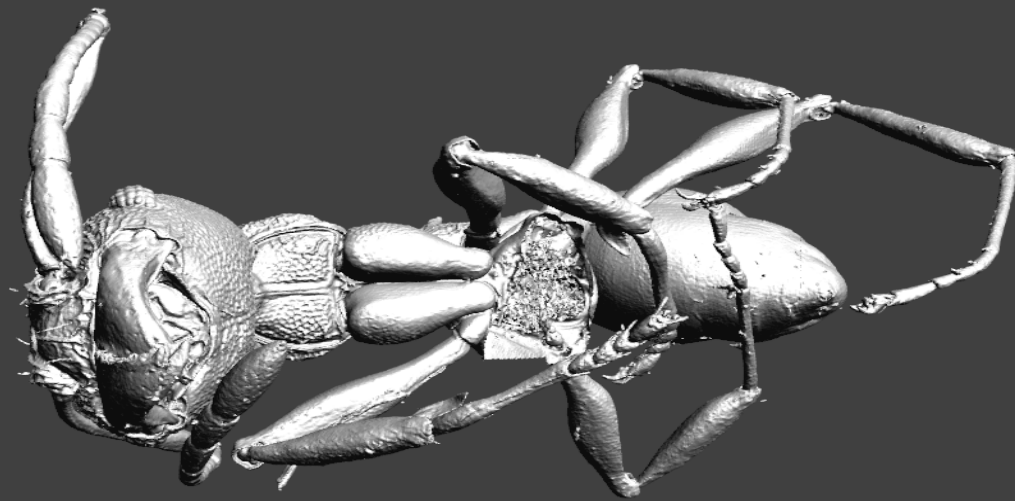

*Pheidole caldwelli*  
minor worker CASENT0709600

Supplement: S2 Fig — (PDF) [file pone.0158544.s002.pdf]

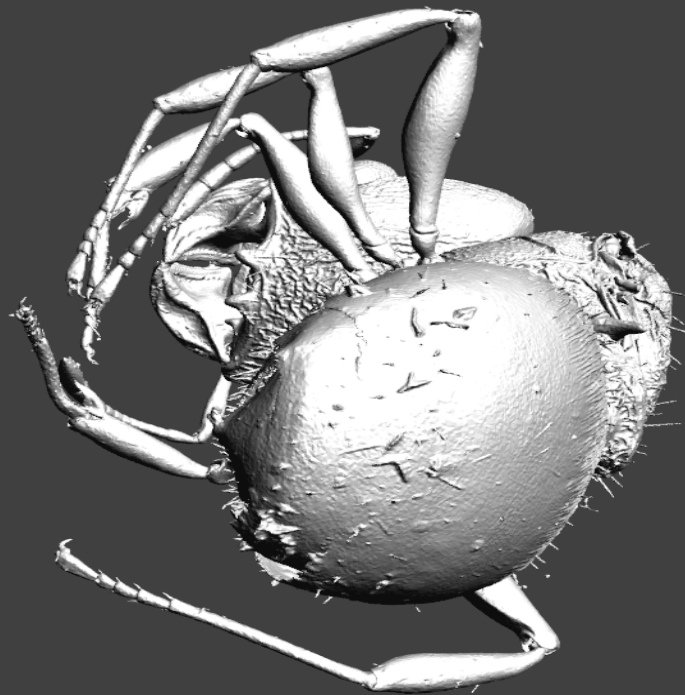

*Pheidole caldwelli*  
queen CASENT0185623

Supplement: S3 Fig — (PDF) [file pone.0158544.s003.pdf]

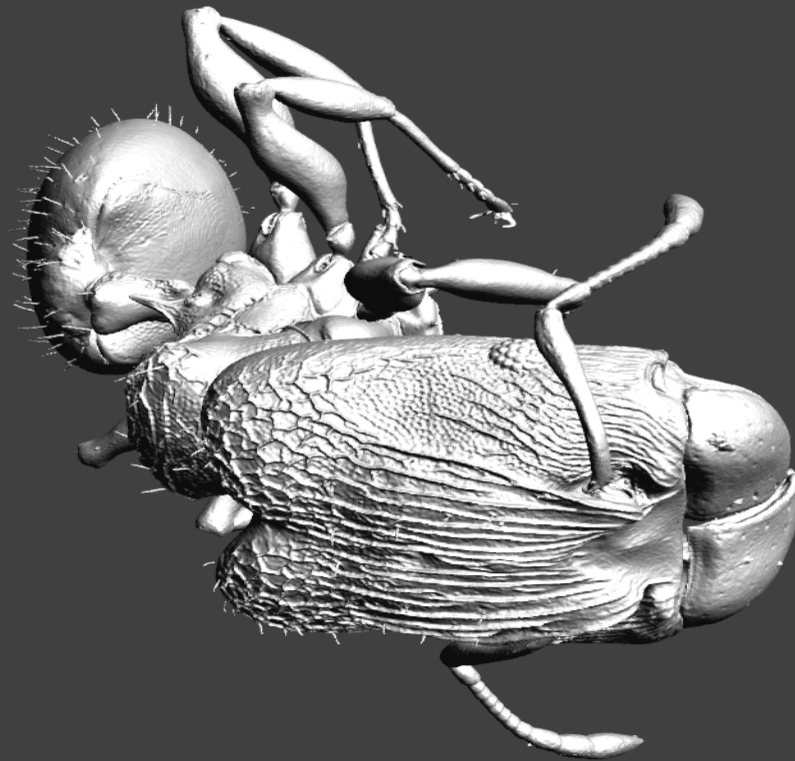

*Pheidole kava*  
major worker CASENT0185437

Supplement: S4 Fig — (PDF) [file pone.0158544.s004.pdf]

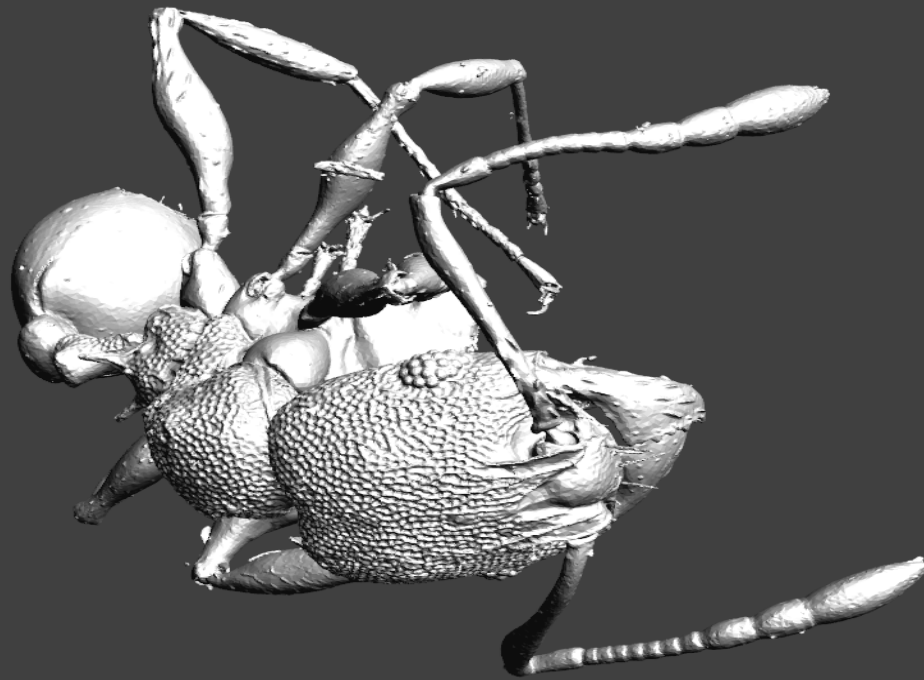

*Pheidole kava*  
minor worker CASENT0183982

Supplement: S5 Fig — (PDF) [file pone.0158544.s005.pdf]

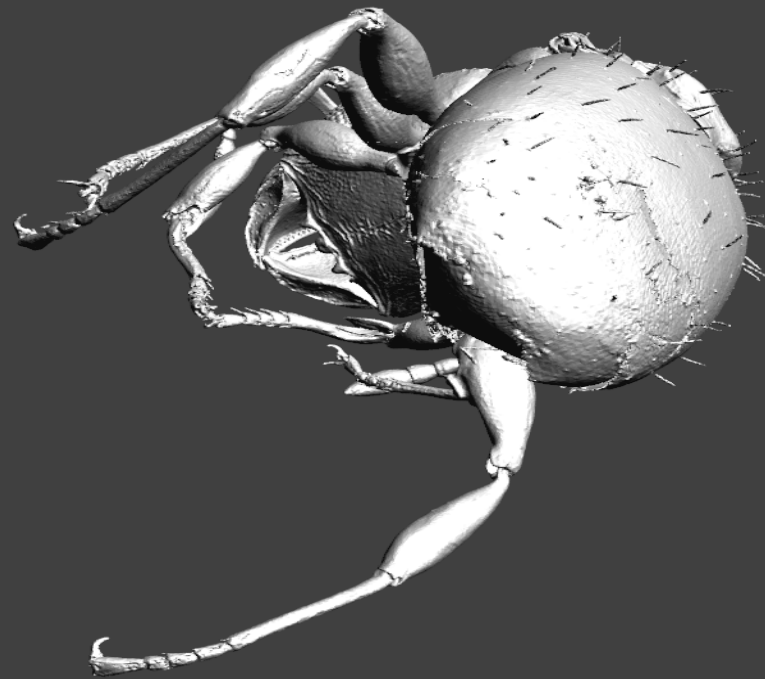

*Pheidole kava*  
queen CASENT0194645

Supplement: S6 Fig — (PDF) [file pone.0158544.s006.pdf]

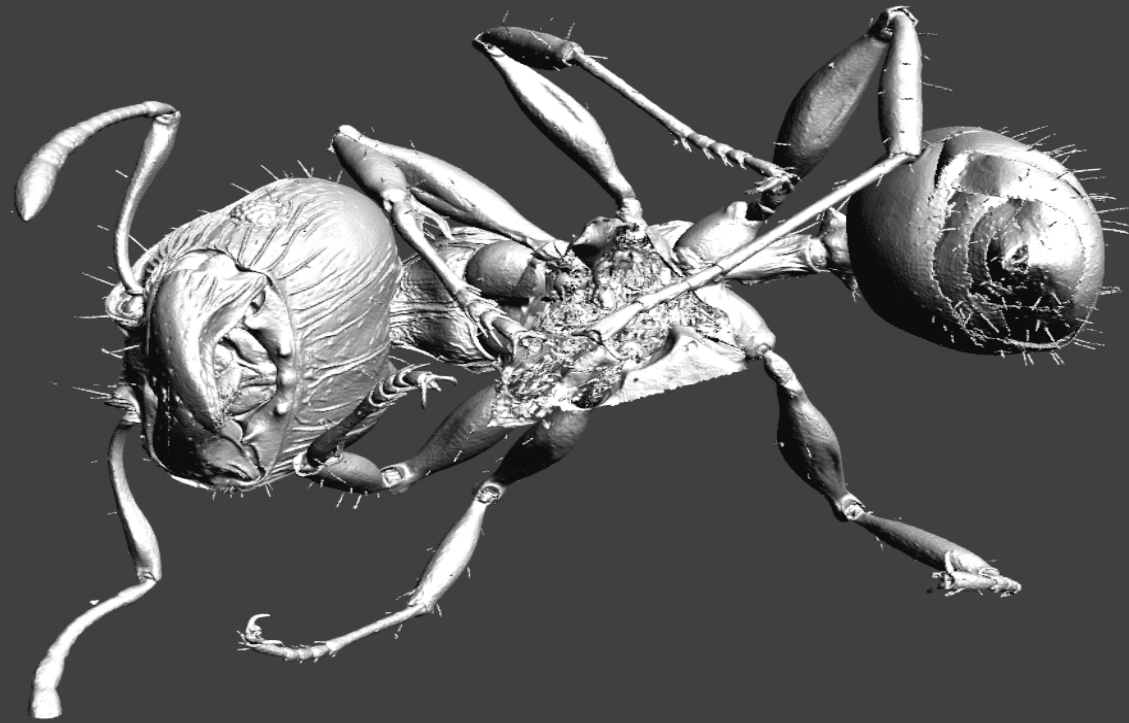

*Pheidole knowlesi*  
major worker CASENT0183992

Supplement: S7 Fig — (PDF) [file pone.0158544.s007.pdf]

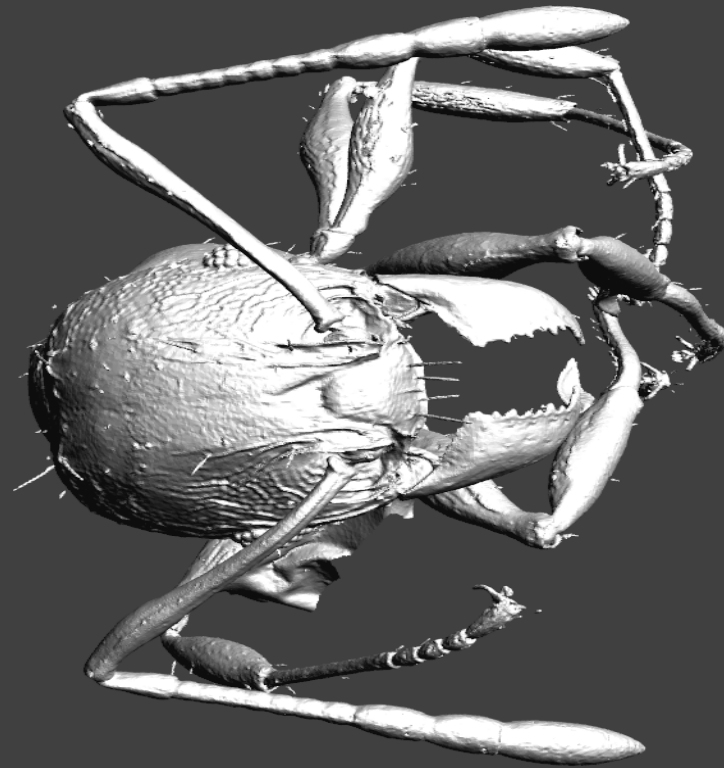

*Pheidole knowlesi*  
minor worker CASENT0184378

Supplement: S8 Fig — (PDF) [file pone.0158544.s008.pdf]

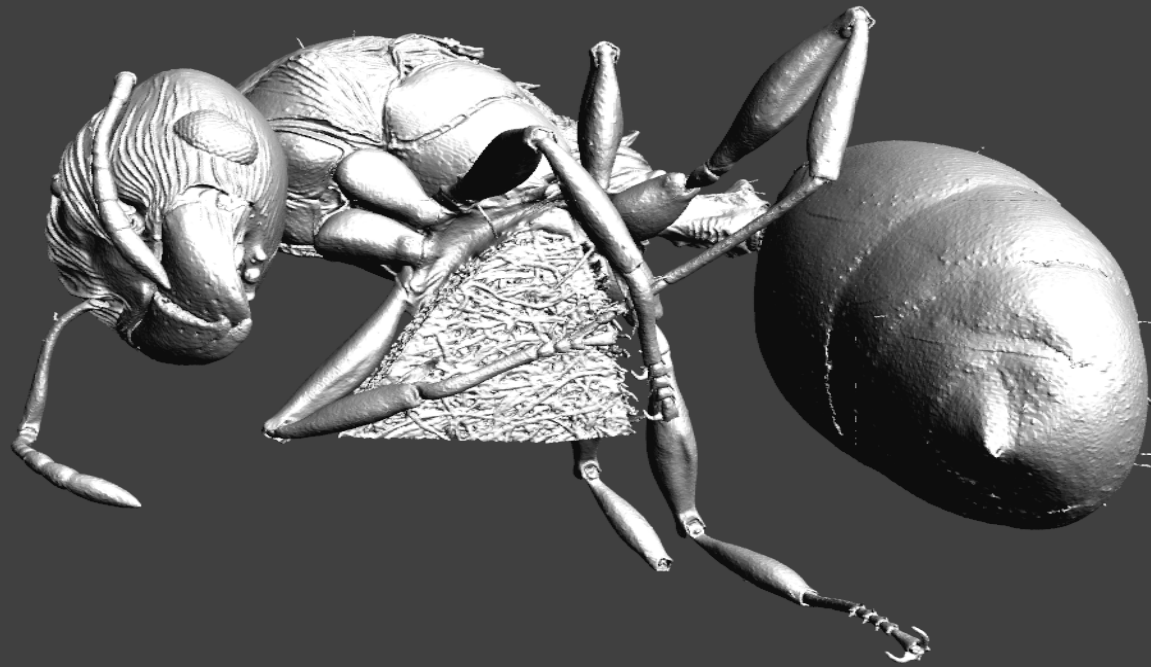

*Pheidole knowlesi*  
queen CASENT0184086

Supplement: S9 Fig — (PDF) [file pone.0158544.s009.pdf]

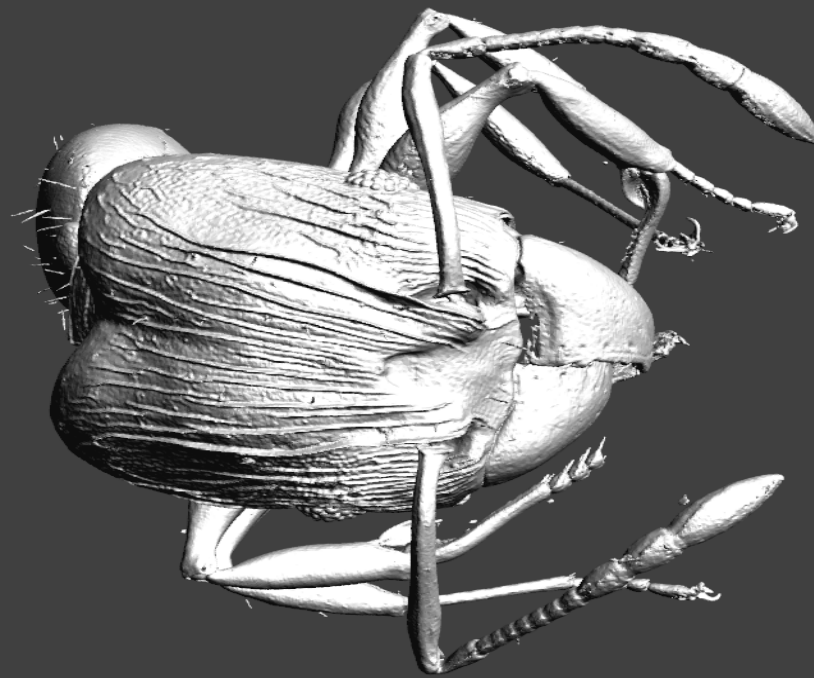

*Pheidole ululevu*  
major worker CASENT0183915

Supplement: S10 Fig — (PDF) [file pone.0158544.s010.pdf]

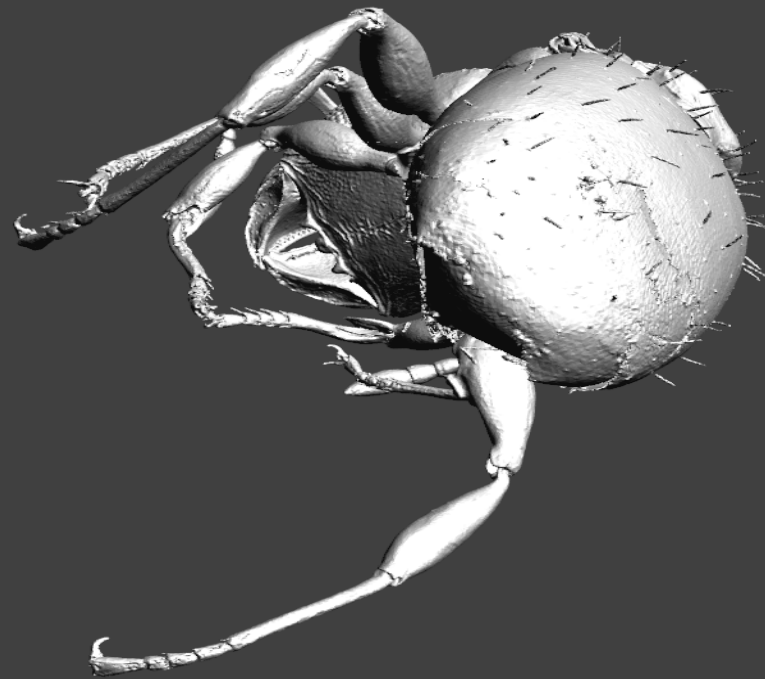

*Pheidole kava*  
queen CASENT0194645

Supplement: S11 Fig — (PDF) [file pone.0158544.s011.pdf]

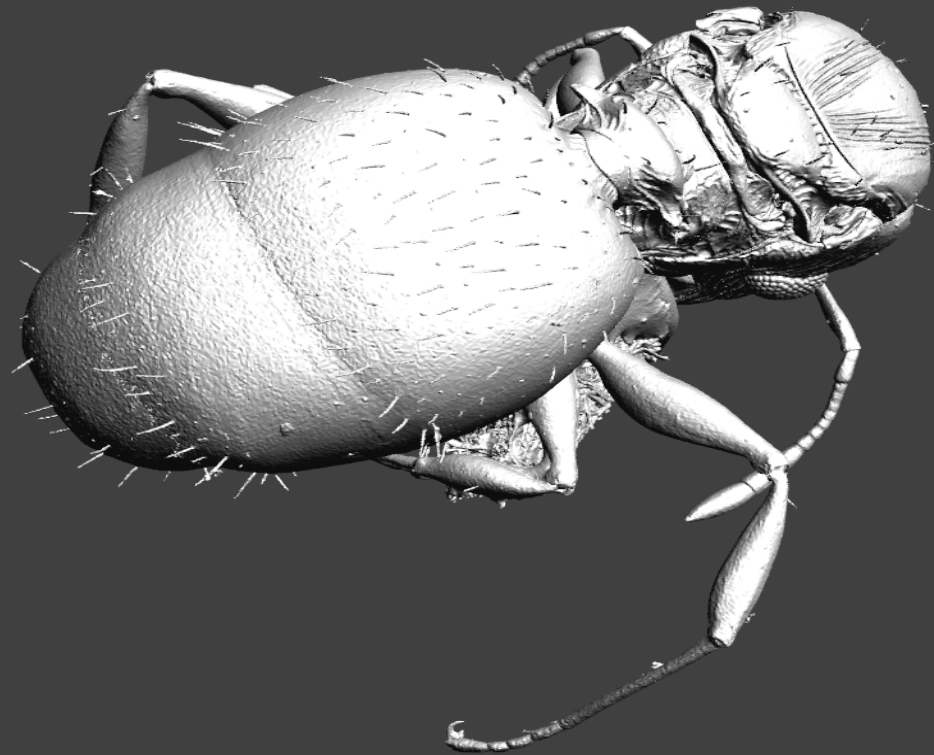

*Pheidole ululevu*  
queen CASENT0185564

Supplement: S12 Fig — (PDF) [file pone.0158544.s012.pdf]

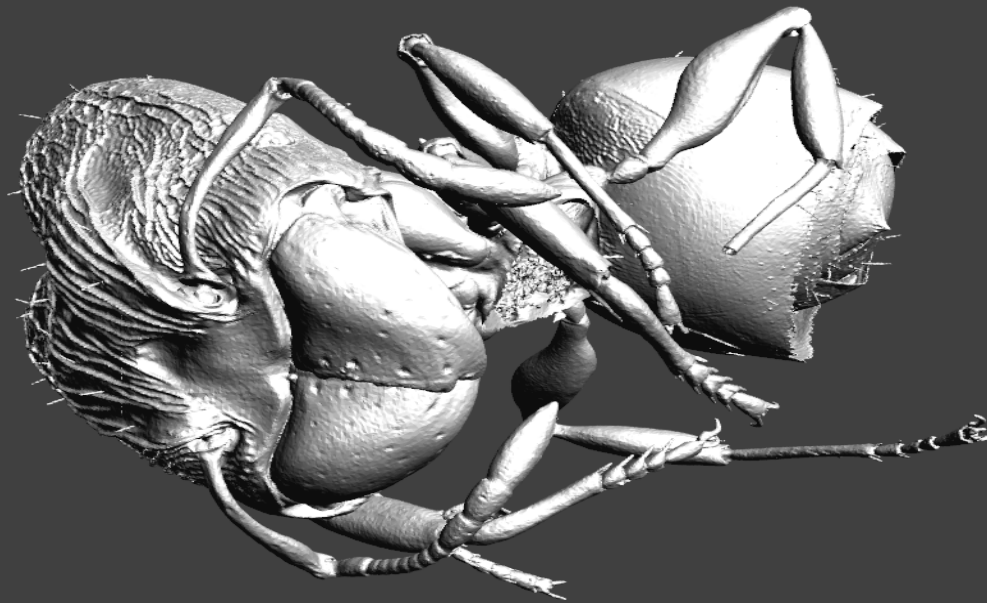

*Pheidole vatu*  
major worker CASENT0184486

Supplement: S13 Fig — (PDF) [file pone.0158544.s013.pdf]

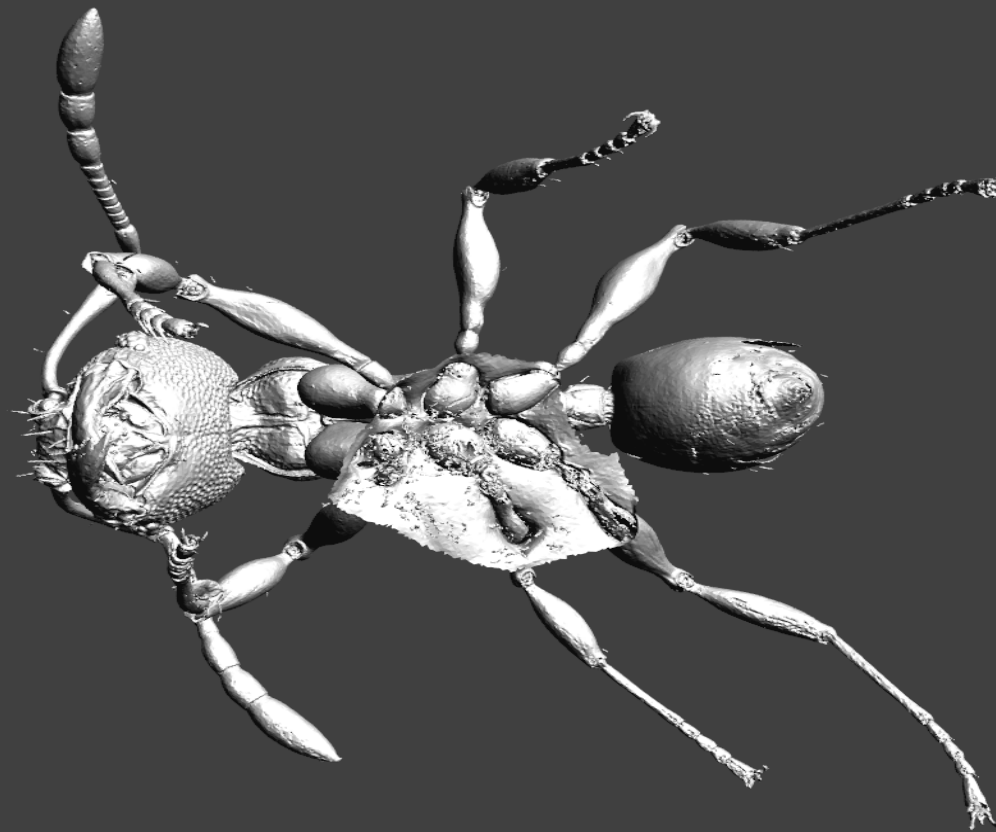

*Pheidole vatu*  
minor worker CASENT0185591

Supplement: S14 Fig — (PDF) [file pone.0158544.s014.pdf]

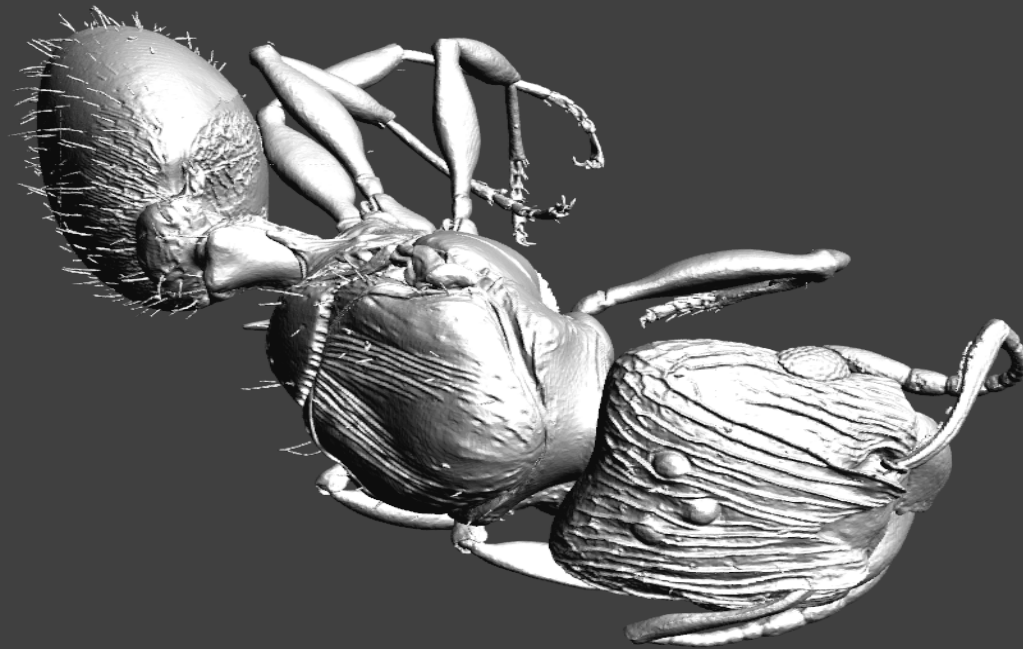

*Pheidole vatu*  
queen CASENT0185452

Supplement: S15 Fig — (PDF) [file pone.0158544.s015.pdf]

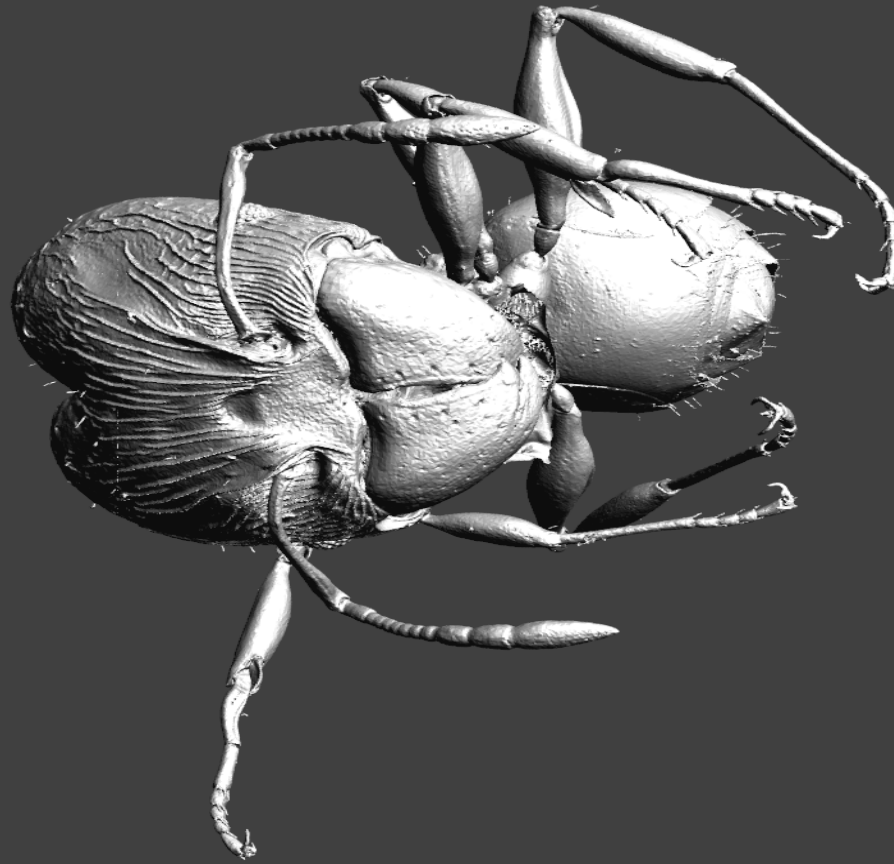

*Pheidole wilsoni*  
major worker CASENT0184311

Supplement: S16 Fig — (PDF) [file pone.0158544.s016.pdf]

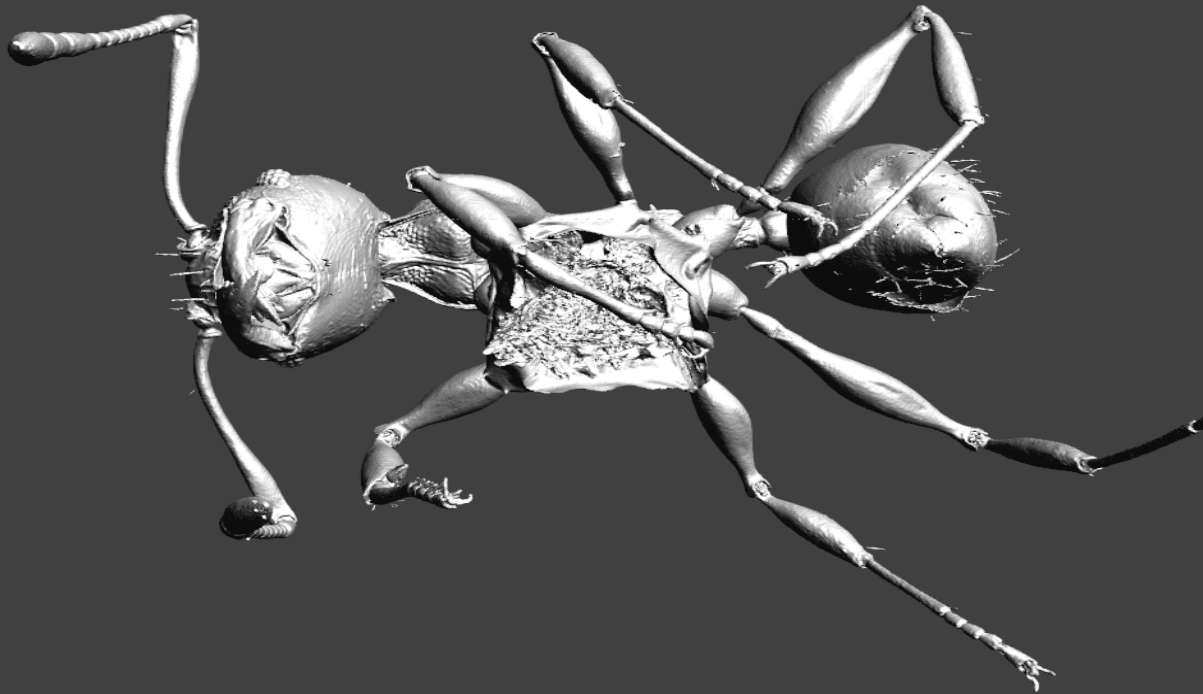

*Pheidole wilsoni*  
minor worker CASENT0184378

Supplement: S17 Fig — (PDF) [file pone.0158544.s017.pdf]

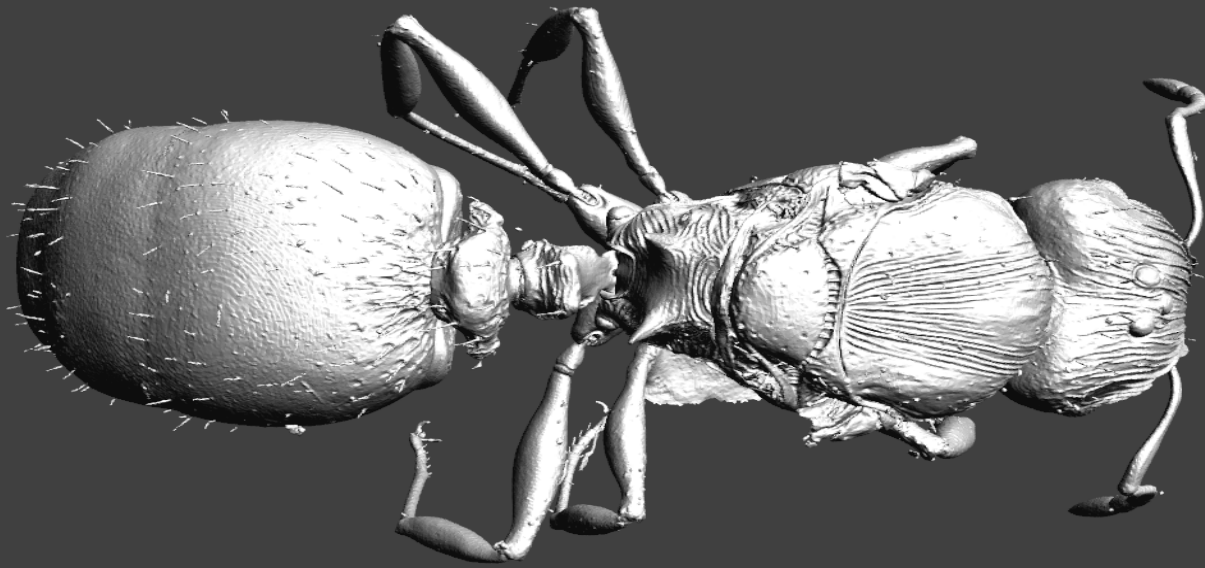

*Pheidole wilsoni*  
queen CASENT0184003

Supplement: S18 Fig — (PDF) [file pone.0158544.s018.pdf]
